# Personalized and Tumor Informed Circulating Tumor DNA Assay for Molecular Residual Disease Monitoring of Solid Malignancies

**DOI:** 10.1002/mco2.70483

**Published:** 2025-12-03

**Authors:** Liping Liu, WenHua Liang, WuQiang Cao, Hongke Wang, HengRui Liang, Liyan Huang, Danman Zhong, Wei Gao, Qiuhua Deng, Yan Zhang, XiaoLing Zeng, Wei Wang, Jun Huang, Chao Yang, GuiLin Peng, XunMei Zheng, JiaXin Ma, XinHua Du, Liang Cui, Yanfang Guan, Jing Bai, Xuefeng Xia, Xin Yi, Jianxing He

**Affiliations:** ^1^ Department of Clinical Laboratory, State Key Laboratory of Respiratory Disease and National Clinical Research Centre For Respiratory Disease The First Affiliated Hospital of Guangzhou Medical University, Guangzhou Medical University Guangzhou China; ^2^ Department of Thoracic Surgery and Oncology, State Key Laboratory of Respiratory Disease and National Clinical Research Centre For Respiratory Disease The First Affiliated Hospital of Guangzhou Medical University, Guangzhou Medical University Guangzhou China; ^3^ Geneplus‐Beijing Institute Peking University Medical Industrial Park, Zhongguancun Life Science Park Beijing China; ^4^ Xiangya School of Pharmaceutical Sciences Central South University Changsha China; ^5^ School of Computer Science and Technology Xi'an Jiaotong University Xi'an China; ^6^ College of Future Technology Peking University Beijing China

**Keywords:** cancer genomics, circulating tumor DNA, minimal residual disease, personalized panel

## Abstract

Emerging evidence suggests that minimal residual disease (MRD) monitoring in solid tumors has implications for prognosis, treatment response, and therapeutic intervention. However, detecting MRD requires highly sensitive and specific circulating tumor DNA (ctDNA) assays. Therefore, we developed an innovative MRD monitoring assay that offers superior performance and cost advantage. Our approach utilizes a comprehensive genomic profiling panel to characterize the patient‐specific mutational landscape of tumor tissue and selects up to 20 top‐ranked variants to design a personalized panel, which is integrated with a tumor‐naive cancer‐type‐specific fixed panel for ultra‐deep sequencing of plasma ctDNA to monitor MRD in common solid tumors. Its limit of detection at the sample level reaches as low as 0.005%, with a specificity of 100%. Furthermore, when applied to colorectal, breast, and lung cancer patients, the ctDNA‐MRD assay accurately predicted postoperative recurrence, prior to radiographic imaging by a median of 112 days for breast cancer and 83 days for lung cancer. In the tracked variants, clonal mutations demonstrated superior prognostic value compared to subclonal variants. This personalized MRD monitoring assay has the potential to enhance early detection of residual or recurrent disease, enable patient prognostic stratification, and inform clinical decision‐making for patients with common solid tumors.

## Graphical abstract

In this study, we developed an innovative ctDNA‐MRD assay that stands out by combining a personalized panel with a fixed panel for specific cancer types. This approach enables the monitoring of MRD through ultra‐deep sequencing of cfDNA in plasma samples. We have also extensively validated the technical performance of this MRD assay and demonstrated its clinical utility in colorectal cancer, breast cancer, and lung cancer.

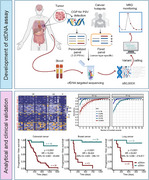

## 1 Introduction

Monitoring of minimal residual disease (MRD) provides a valuable opportunity to predict recurrence for cancer patients on the basis of an occult or persistent state of disease [1, 2]. The timely intervention or modification of treatment decisions informed by MRD has the potential to increase patient benefit and minimize treatment‐related toxicity.

The current routine clinical approach for monitoring recurrence is serial radiographic imaging, which is limited by its risk of radiation exposure, and the requirement of specialized equipment [3]. Moreover, MRD can persist at levels below the detection threshold of imaging and eventually result in relapse [4]. Therefore, there is an urgent clinical need for more sensitive and convenient MRD detection for recurrence monitoring after curative‐intent therapy, thereby offering the potential for early intervention, ultimately aiming to improve the cure rate and prolong survival for cancer patients.

At present, monitoring MRD by tracking ultra‐low‐frequency somatic mutations in circulating tumor DNA (ctDNA) has shown promising prospects in the management of cancer patients [2]. Studies have shown that ctDNA can noninvasively, dynamically, and in real‐time reflect patients' MRD and tumor burden [2]. Increasing investigations have established clinical associations between MRD and clinical outcomes. It is widely recognized that patients with detectable MRD after surgery or curative chemoradiotherapy have a worse prognosis, a higher risk of recurrence, and are more likely to benefit from adjuvant therapy than those with undetectable MRD [3, 5, 6, 7, 8, 9]. Numerous studies have also demonstrated that ctDNA‐MRD can hint tumor recurrence months earlier than imaging modalities, providing a valuable time window for clinical intervention [3, 5, 7, 8].

However, the clinical utility of MRD highly relies on the variation of ctDNA detection methodologies (such as DNA input and sequencing depth) [10] and analytical validation measures (such as sensitivity and specificity) [2]. Fixed panel assays are designed using large genomic datasets from cancer populations, detecting driver genes, frequently mutated genes, and genes associated with drug resistance and tumor evolution. These fixed‐panel assays are off‐the‐shelf tests, using the same panel for all patients, such as CAPP‐seq [11], allowing for convenient and rapid clinical application and monitoring of the acquired mutations resulting from tumor evolution. However, fixed‐panel assays suffer from limited coverage of patient‐specific mutations and are constrained by cost considerations, leading to lower sequencing depth. Consequently, the sensitivity of MRD detection may be compromised [3]. Additionally, they are inapplicable to certain rare cancer types in which the mutations are not included in pre‐designed panels. In contrast, bespoke MRD assays, such as Signatera [12] and the Invitae Personalized Cancer Monitoring assay using patient‐specific anchored‐multiplex PCR (AMP) technology [13, 14, 15], can exclusively detect the mutations known to present in the tumor using a deeper sequencing, thus increasing the sensitivity for detecting truly tumor‐derived mutations despite low frequency [5]. However, personalized assays also have shortcomings, as they can be affected by spatial and temporal tumor heterogeneity and are unable to monitor emerging mutations in resistant subclones. Furthermore, personalized assays often require prior whole‐exome sequencing (WES) of the tumor [3], which is known for its exceptional performance but can be costly. Fortunately, a recent study has demonstrated that a tumor comprehensive genomic profiling (CGP)‐informed personalized ctDNA assay has achieved comparable results to WES with significantly lower cost in detecting patient‐specific alterations for MRD monitoring of bladder cancer patients [16].

In this study, we present an innovative ctDNA‐MRD assay that combines the advantages of both fixed and personalized panels. It involves initially using a CGP to detect patient‐specific variant signatures of tumor tissues, followed by the selection of 2–20 mutations for the design of a personalized panel. Our MRD monitoring assay stands out by combining this personalized panel with a fixed panel for specific cancer types, which includes recurrent mutations with high evidence level, for the monitoring of MRD by ultra‐deep sequencing (≥80,000x) of ctDNA in plasma samples. We have also extensively validated the technical performance of this MRD assay and demonstrated its clinical utility in multiple cancer types.

## 2 Results

### 2.1 Overview of the ctDNA‐MRD Assay

**FIGURE 1:**
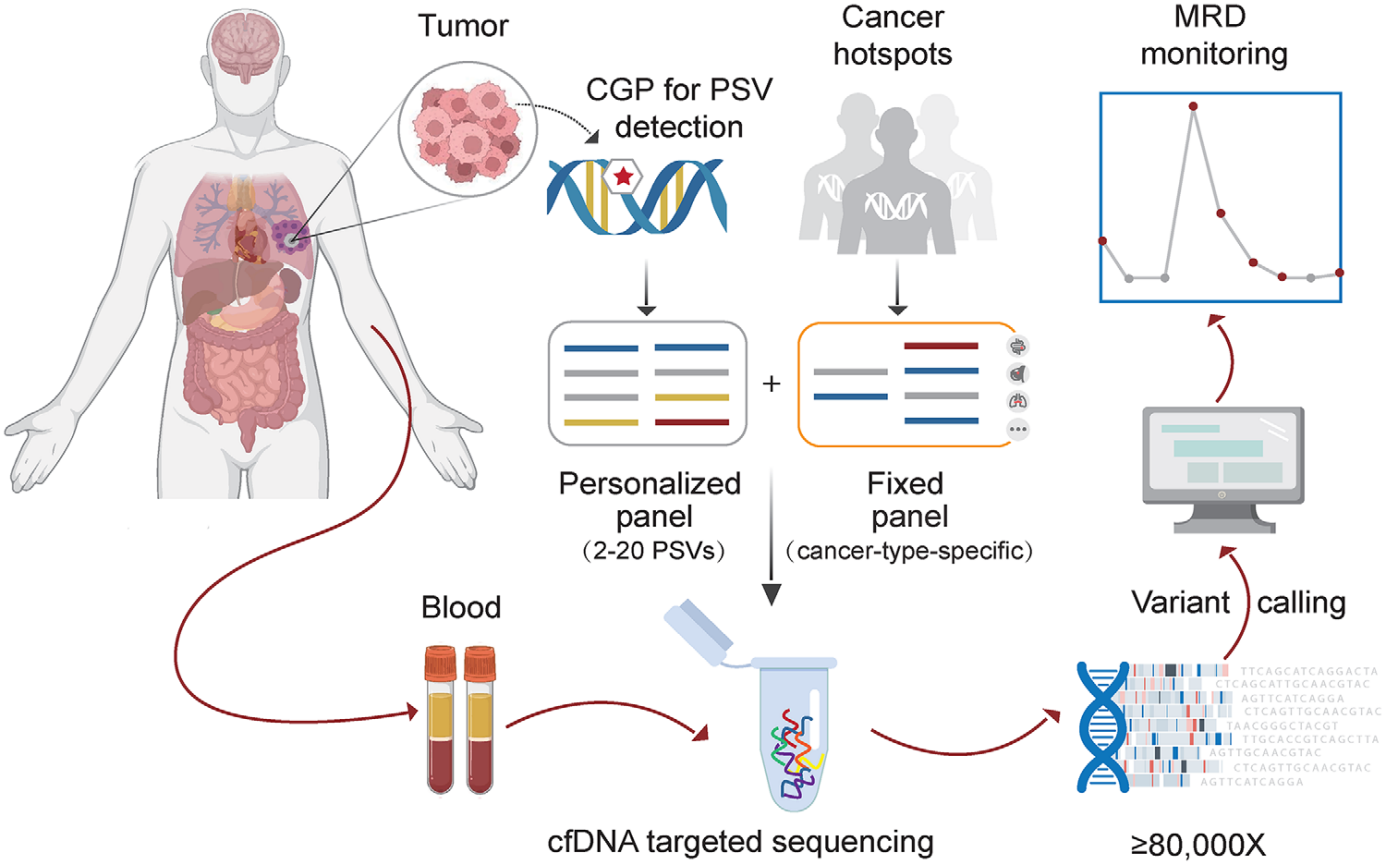
Workflow of the ctDNA‐MRD assay. This ctDNA‐MRD assay utilizes a 1021‐gene panel for comprehensive genomic profiling (CGP) of patients' tumor tissue, enabling the design of tumor‐informed personalized panel for MRD monitoring. The 1021‐gene panel is pre‐designed from our analysis of over 350,000 tumor tissue samples, encompassing genes associated with tumor initiation, progression, and targeted therapy or immunotherapy. The tumor‐naive fixed panel is pre‐designed based on the cancer‐type specific mutation hotspots and clinically actionable mutations. Plasma cfDNA samples are subjected to ultra‐deep (≧80,000x) targeted sequencing for ctDNA detection using both the personalized panel and cancer‐type specific fixed panel. This is complemented by rigorous data quality control and ctDNA somatic mutation calling algorithm, facilitating the ctDNA‐MRD monitoring of patients with common solid tumors.

We illustrated the workflow of our ctDNA‐MRD assay in Figure 1. Detailed descriptions can be found in Section 5. It is worth noting that we employed a fixed 1021‐gene panel for the CGP of tissue samples to detect tumor‐derived mutations, which allowed us to design patient‐specific personalized panels, and, meanwhile, provide a cost‐effective alternative to WES. What sets our ctDNA‐MRD assay apart from other methods is the integration of both personalized panel and cancer‐type‐specific panel. The personalized panel enables sensitive monitoring of tumor‐derived mutations, while the cancer‐type‐specific fixed panel can detect the emergence of new/resistant mutations, thus partially mitigating the spatiotemporal heterogeneity of tumors and providing valuable insights for clinical decision‐making of targeted therapy. Additionally, the utilization of fixed panels also allows for quality control of ctDNA‐MRD detection experiments. Up to now, we have developed four cancer‐type specific fixed panels for lung cancer (LC), colorectal cancer (CLC), breast cancer (BC), and pan‐cancer (esophageal cancer, pancreatic cancer, ovarian cancer, endometrial cancer, gastric cancer, urothelial carcinoma, etc.). These fixed panels could cover most of the common solid tumor patients (Figure S1), making this assay a powerful tool for ctDNA‐MRD monitoring in a wide range of solid tumor types.

### 2.2 Development of ctDNA‐MRD Assay

The establishment of our ctDNA‐MRD assay begins with determining the input amount of cell‐free DNA (cfDNA). We inferred with a binomial distribution method that when the limit of detection (LoD) of a single mutation reaches 0.1%, an effective depth of at least 3000x is required (Figure S2A). To ensure this, the depth requirement is met across all samples; a DNA input of ≥30 ng is sufficient (Figure S2B). By increasing the DNA input to ≥60 ng, we can achieve an effective depth of ≥8000x for most samples (85.7% for samples with 60–80 ng DNA input, Figure S2B). As the DNA input further exceeded 80 ng, the effective depth reached saturation and subsequently exhibited a declining trend (Figure S2B). Therefore, we determined a starting DNA input of 30 ng as the minimum requirement, while 80 ng as the maximum DNA input. We also analyzed the cfDNA quantities of 1173 samples, where two tubes of blood (approximately 20 mL) were utilized for cfDNA extraction. Nearly, all the samples (97.6%) yielded cfDNA quantities more than 30 ng, while 61.4% of the samples exhibited cfDNA quantities more than 60 ng. To ascertain the sequencing depth for ctDNA‐MRD assay, we conducted a saturation analysis using five plasma samples with a starting amount of 80 ng DNA. We found that the duplication rate exceeded 80% when the raw sequencing depth reached 80,000× (Figure S2C). Based on this finding, we set the raw sequencing depth of 80,000× as one of our quality control criteria in ctDNA‐MRD assay. These results indicate that the ctDNA‐MRD assay can be established with minimal 30 ng, maximum 80 ng, and 80,000× raw sequencing depth.

### 2.3 LoD at Variant Level

**FIGURE 2:**
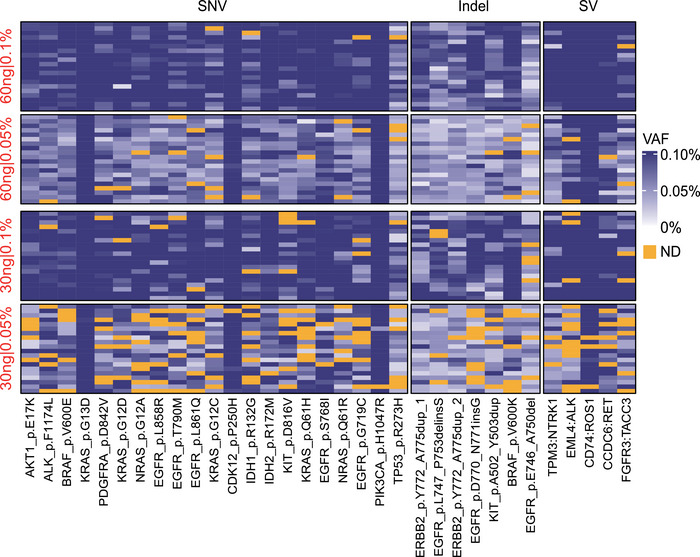
Detection of variants with 0.1% and 0.05% VAFs. The heatmap presented measured VAFs of 33 monitored variants (21 SNVs, 7 InDels, and 5 SVs) in 20 standard samples. The theoretical VAFs (0.1% and 0.05%) and DNA input (60 and 30 ng) were annotated on the left side of the heatmap. Each row represents a sample, while each column represents a monitored variant. The gold color represents undetected variants. ND, not detected.

The LoD is a crucial parameter that defines the lowest level at which a genomic variant can be consistently detected, ensuring a high detection rate of 95% [17]. In order to assess the LoD of our ctDNA‐MRD assay for different variant types, we employed the ctDNA standard sample. This standard sample carries 21 single nucleotide variants (SNVs), seven insertions and deletions (InDels), and five structural variants (SVs), covering a wide range of common hotspot or clinically actionable mutations in lung, colorectal, breast, and other cancer types. Notable examples include *EGFR* p.L858R, *BRAF* p.V600E, and *PIK3CA* p.H1047R. All mutations are listed in Table S1. We analyzed a total of 20 samples with 60 ng and the minimal DNA input of 30 ng for five VAF settings. The detection results of 0.1% and 0.05% VAFs were illustrated in Figure 2, while results of 0.02%, 0.01%, and 0.005% VAFs are shown in Figure S3. Remarkably, when the DNA input was 60 ng and the VAF was 0.1%, nearly all mutations were detected with high sensitivity (SNVs: 99.0%; InDels: 100%; SVs: 99.0%, Figure 2, Figure S4). When the VAF was reduced to 0.05%, the positive percent agreement (PPA) for SNVs, InDels, and SVs remained above 95% (SNVs: 95.5%; InDels: 95.7%; SVs: 96.0%, Figure 2, Figure S4). Thus, we can conclude that the LoD of our ctDNA‐MRD assay for site‐level SNVs, InDels, and SVs is 0.05% when the DNA input is 60 ng. Even for variants with a VAF as low as 0.02%, our assay demonstrates a high positive detection rate for SNVs (82.4%), InDels (79.3%), and SVs (70.0%) (Figure S4). In addition, the LoD for SNVs, InDels, and SVs in the assay has been determined to be 0.1% at the minimal DNA input of 30 ng (Figure 2, Figure S4B, D). These findings underscore the robustness and exceptional sensitivity of our ctDNA‐MRD assay in detecting low‐frequency variants of SNV, InDel, and SV.

### 2.4 LoD at Sample Level

**FIGURE 3:**
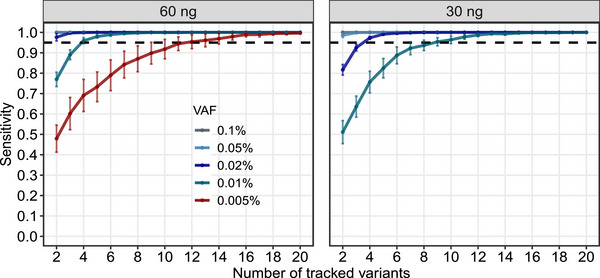
Sample‐level MRD detection with 2–20 tracked variants. The sample‐level sensitivity of the ctDNA‐MRD assay at different VAFs of 0.1%, 0.05%, 0.02%, 0.01%, and 0.005% was assessed using 2–20 tracked variants. The black dashed line represents the 95% sensitivity threshold. Error bars depict the mean sensitivity ±95% confidence interval. DNA inputs of 60 and 30 ng were illustrated separately.

For ultra‐low frequency ctDNA variants with a VAF of < 0.1%, increasing the number of tracked mutations can enhance the sensitivity of MRD detection at the same DNA input. Therefore, to assess the LoD of sample‐level ctDNA detection, we randomly sampled *N* mutations (*N* = 2, 3, …, 20) from the detection results of the aforementioned 20 standard samples. We repeated this sampling process 100 times and then calculated the sensitivity of MRD detection when monitoring N mutations. As shown in Figure 3, with a DNA input of 60 ng, we observed a remarkable sample‐level LoD of 0.02% (with a sensitivity of 97.6%) for monitoring two mutations. Notably, the sample‐level LoD further decreased to 0.01% when monitoring 4–11 mutations, and 0.005% when monitoring 12–20 mutations. Decreasing the DNA input to 30 ng resulted in a sample‐level LoD of 0.05% for monitoring two mutations, 0.02% for monitoring four to eight mutations, and 0.01% for monitoring more than nine mutations. These findings aligned with previous reports that higher DNA input leads to lower sample‐level LoD, and monitoring more mutations also contributes to a reduced sample‐level LoD [18, 19]. The ability to accurately detect low‐frequency variants with superior sample‐level LoD highlights the potential of our assay as a valuable tool for ctDNA‐MRD monitoring.

### 2.5 Specificity of Variant and Sample Level

The limit of blank (LoB) is the highest measurement result likely to be observed for a blank sample with a false positive rate of 0.05^17^. In this study, we evaluated the LoB of this ctDNA‐MRD assay using the aforementioned DNA standard sample and cfDNA samples from healthy individuals. For the variant‐negative standards, we tested 20 samples with 80 ng DNA input for each sample. Among the 660 negative loci monitored in all 20 samples, only one SNV locus yielded a false positive call, resulting in a negative concordance rate of 99.8% (419/420) for SNVs, 100% (140/140) for InDels, and 100% (100/100) for SVs, and the overall negative percent agreement (NPA) was 99.85% (659/660) across the variant types. Next, we recruited 22 healthy donors, with each providing two tubes (∼20 mL) of blood sample. For each healthy donor, we randomly selected five panels from the personalized panels of 35 cancer patients, with each panel containing 20 PSVs, resulting in a total of 100 true negative mutations detected per cfDNA sample. Out of the total 2200 negative loci monitored, only five loci exhibited false positive results, resulting in an overall NPA of 99.8% (2195/2200). Therefore, whether using variant‐negative standards or normal cfDNA samples, the variant‐level LoB of our ctDNA‐MRD assay remains at 0% with a specificity of 99.5%. We have integrated the variant‐level specificity results from both the standards and healthy donors and presented them in Figure S5A. Furthermore, the sample‐level specificity remained at 100% when monitoring 2–20 mutations (Figure S5B). These results highlight the outstanding reliability of the ctDNA‐MRD assay in accurately identifying true negative results while maintaining a low false positive rate.

### 2.6 Accuracy of ctDNA Quantification

**FIGURE 4:**
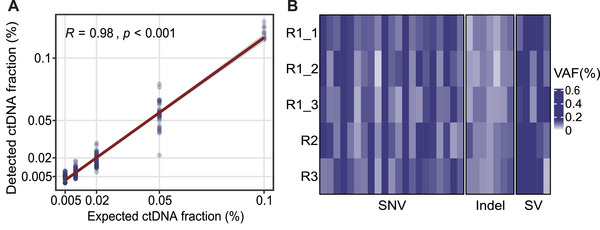
Accuracy and precision analysis of the ctDNA assay. (A) The expected ctDNA fraction in SW480 cell line DNA standards was well correlated (Pearson correlation) with the ctDNA fraction detected by the ctDNA‐MRD assay. The accuracy of ctDNA quantification were measured with ctDNA fractions of 0.1%, 0.05%, 0.02%, 0.01%, and 0.005%. (B) The heatmap showed VAFs of all 33 variants (21 SNVs, 7 InDels, and 5 SVs) obtained from five tests. Among them, R1_1, R1_2, and R1_3 were performed by the same operator in a single experiment, while R2 and R3 were independently conducted by two other operators. The DNA input was 60 ng. The darker color of blue indicates higher VAFs. VAF, variant allele frequency.

We employed mean tumor molecules (MTM) per milliliter of plasma to quantify tumor‐specific variants for patients with positive ctDNA calls. This absolute quantification approach takes into account not only the ctDNA fraction but also the cfDNA quantity (ng per mL of plasma), thereby providing a more accurate estimation of ctDNA levels compared to relative quantification methods. However, accurate determination of ctDNA fraction remains crucial for the accurate calculation of MTM. To assess the reliability of our assay in ctDNA quantification, we employed SW480 cell line DNA standards with ctDNA fractions of 0.1%, 0.05%, 0.02%, 0.01%, and 0.005% to assess the consistency between the expected and measured DNA content. The results demonstrated a high level of consistency between the expected and the detected ctDNA fraction by our ctDNA‐MRD assay, with a *R* value of 0.98 (Pearson correlation, *p* < 0.001, Figure 4A), highlighting its exceptional accuracy in ctDNA quantification.

### 2.7 Stability of ctDNA Quantification

To assess the impact of exogenous and endogenous interferents on the accuracy of ctDNA detection, we conducted verification experiments to examine the effects of three common interferents, namely, hemoglobin, isopropanol, and ethanol, on the ctDNA‐MRD assay. Five groups were established, including “no interferent control,” “2 mg/mL hemoglobin,” “1% isopropanol,” “1% ethanol,” and “2% ethanol.” The verification employed commercially available standard samples with a mutation frequency of 0.1%, and each group was repeated three times with 30 ng DNA input. The results demonstrated that the average mutation detection rates of the positive standard samples were >97% (97.6%–100%) in both the control group without interferents and the experimental groups with added interferents (Figure S6). No significant differences were observed among these groups, indicating minimal impact of interferents on ctDNA mutation detection.

### 2.8 Precision Analysis: Repeatability and Reproducibility

Precision refers to the consistency of measurement results obtained from multiple analyses of the same sample, encompassing both repeatability (intra‐run) and reproducibility (inter‐run) [17]. In this study, we also evaluated the precision of the ctDNA‐MRD assay using the commercially available MRD standard sample mentioned above, with a VAF of 0.1%. A total of five replicate tests were conducted to assess the repeatability and reproducibility of our assay. Among these, three replicates were performed by the same operator, and sequenced in the same run, to evaluate repeatability. The remaining two replicates were prepared by two different operators in two separate batches and sequenced in different runs, aiming to assess reproducibility. When DNA input was 60 ng, all 33 variants were consistently detected as positive calls in all five tests, resulting in a detection concordance rate of 100%. The VAFs of all 33 variants obtained from the five tests are displayed in Figure 4B. Using 30 ng of DNA as input, one InDel (*KIT* p.A502_Y503dup) was undetected in one of the five replicates, providing a PPA of 99.4% (159/160) for mutation detection (Figure S7). These findings highlight the excellent repeatability and reproducibility of the ctDNA assay in variant detection and the utilization of MRD monitoring.

### 2.9 Prediction of Recurrence Risk for Cancer Patients

Following curative treatment, monitoring MRD through ctDNA assay can provide valuable insights into cancer recurrence risk and guide therapeutic decisions. To assess the performance of our assay in predicting recurrence risk in real‐world clinical samples, we retrospectively analyzed ctDNA‐MRD in a cohort of 46 patients with CLC who underwent curative treatment (Figure S8). The median age at diagnosis was 58 (34–84) years. Among these 46 patients, 27 (58.7%) were male and 19 (41.3%) were female. The distribution of disease stages was as follows: six (13%) patients were classified as stage I, 12 (26.1%) as stage II, 22 (47.8%) as stage III, and six (13%) as stage IV (Table S2). The median follow‐up duration for these patients was 587 (65–1651) days.

**FIGURE 5:**
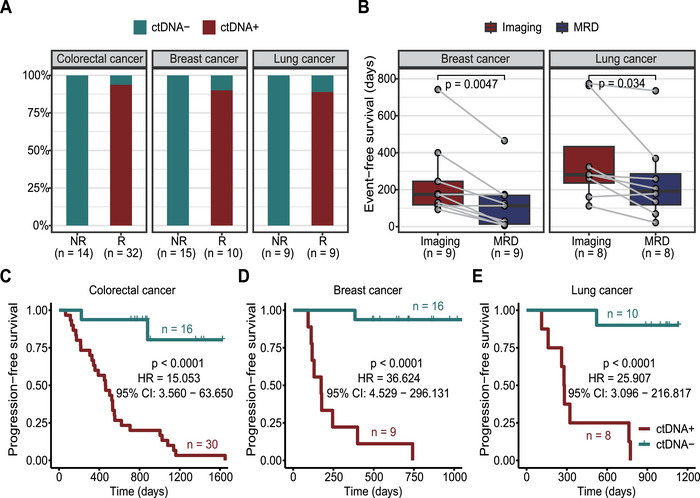
Validation of the ctDNA‐MRD assay used for predicting cancer recurrence. (A) The proportion of patients with detectable MRD (ctDNA+) and undetectable MRD (ctDNA−) was assessed among patients (46 CRC, 25 BC, and 18 LC) with postoperative recurrence (R) and without recurrence (NR). (B) Paired comparison of the recurrence‐free survival for patients with postoperative relapse defined by imaging or ctDNA‐MRD. The recurrence‐free survival of (C) CRC, (D) BC, and (E) LC patients, stratified by their ctDNA status (ctDNA+ vs. ctDNA−). MRD, minimal residual disease; CRC, colorectal cancer; BC, breast cancer; LC, lung cancer.

We performed CGP on tumor samples and successfully devised a personalized monitoring panel with 2–20 PSVs for each of the 46 CRC patients (the median number of PSV = 10, Figure S9A). By integrating the personalized panel with the fixed panel for CLC, we conducted ctDNA detection using plasma samples collected after surgery for MRD monitoring (Figure S9A). The median cfDNA concentration was 11.6 ng (Q1: 10.4, Q3: 20.2) per milliliter of plasma. And the cfDNA samples were sequenced with a median raw depth of 166,396× (Q1: 138,541, Q3: 232,351), while the median depth of effective reads was 8227× (Q1: 4997, Q3: 11,616) (Figure S10). During the follow‐up period, 32 patients experienced recurrence, of which 30 were MRD positive (Figure 5A, S8). The predictive sensitivity for recurrence detection was determined to be 93.8% (30/32). We illustrated the estimated VAF, ctDNA fraction, and MTM of CRC patients with detectable MRD in Figure S9. On the other hand, 14 patients maintained a recurrence‐free status until the end of the follow‐up, with all their ctDNA‐MRD tests yielding negative results, indicating a specificity of 100% (Figure 5A, S8). Kaplan–Meier plots revealed that patients who test positive for MRD exhibit a higher risk of recurrence compared to those who test negative for MRD (log‐rank test, *p* < 0.0001, HR = 15.053, 95% CI: 3.56–63.65) (Figure 5C), aligning with previous reports on MRD in CLC [20, 21].

We further collected postoperative plasma samples from 25 triple‐negative BC patients to validate the prognostic value of the ctDNA assay in MRD monitoring (Figure S11). The patients had a median age of 44 (28–73) years. We collected median 2 (range:1–4) postoperative plasma samples for each of them, and the median follow‐up was 644 days (range: 93–1102) (Table S3). The cfDNA concentration, raw, and effective sequencing depth are shown in Figure S10. MRD was tracked with a median of 6 (2–18) PSVs (Figure S12A) and the BC‐specific fixed panel. A total of 10 patients experienced postoperative recurrence, among whom nine patients had at least one positive detection of ctDNA in postoperative blood samples (Figure 5A, S11). In the observed cases, MRD was detected prior to radiographic evidence of disease recurrence in seven out of the nine (77.8%) BC patients (Figure S11), providing a median lead time of 112 days between MRD detection and imaging‐confirmed clinical recurrence. (Figure 5B). The VAF, ctDNA fraction, and MTM of patients with detectable ctDNA are shown in Figure S12. Conversely, all 15 patients without recurrence tested negative for MRD (Figure 5A, S11). Therefore, the sensitivity and specificity of MRD prediction for BC recurrence were 90% (9/10) and 100% (15/15), respectively. BC patients who consistently maintained MRD negative after surgery exhibited significantly longer progression‐free survival compared to those who had ever tested positive for MRD (log‐rank test, *p* < 0.0001, HR = 36.624, 95% CI: 4.529–296.131) (Figure 5D).

In the LC cohort with 18 patients, the median age of diagnosis was 59 (37–79), and half of the patients (9/18) were female (Table S4). They were followed up for median 832 (112–1134) days. A median of 2 (1–5) postoperative plasma samples were collected for each LC patient (Figure S13). We displayed the quality control of cfDNA concentration and sequencing depth in Figure S10. The ctDNA‐MRD was monitored using personalized panel including a median of 7 (3–20) PSVs (Figure S14A) along with the fixed panel for LC. We presented the recurrence status of patients and their MRD monitoring history in Figure S13. During the follow‐up period, nine patients experienced recurrence. Among them, eight patients tested positive for MRD after surgery (Figure 5A, Figure S13), and seven of them (87.5%) had MRD detected prior to the image‐confirming recurrence, with a median lead time of 83 days (Figure 5B, Figure S13). We also presented the VAF, ctDNA fraction, and MTM per milliliter of plasma in Figure S14. All nine patients who did not experience recurrence during follow‐up had negative MRD test results (Figure 5A, Figure S13), resulting in a sensitivity of 88.9% (8/9) and a specificity of 100% (9/9) for MRD prediction of LC recurrence. Similarly, patients who consistently maintained MRD undetectable after surgery exhibited significantly longer disease‐free survival compared to those who had detectable MRD (log‐rank test, *p* < 0.0001, HR = 25.907, 95% CI: 3.096–216.817) (Figure 5E). These findings provide compelling evidence that our assay can accurately predict the risk of recurrence and the detection of MRD‐preceded radiographic progression.

### 2.10 Monitoring of MRD With Clonal and Subclonal Mutations

**FIGURE 6:**
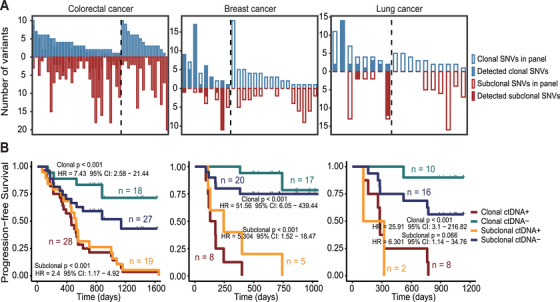
Comparing prognostic value of ctDNA‐MRD tracked with clonal and subclonal mutations. (A). Bar plots illustrated the number of clonal and subclonal mutations included in each patient's personalized panel and the detected ctDNA clonal and subclonal mutations in patients with CRC, BC, and LC. Patients with (left) or without (right) detectable ctDNA were separated by a black dashed line. (B) The recurrence‐free survival of CRC, BC, and LC patients, stratified by the detection of ctDNA clonal mutations (clonal ctDNA+ vs. clonal ctDNA−) or subclonal mutations (subclonal ctDNA+ vs. subclonal ctDNA−). CRC, colorectal cancer; BC, breast cancer; LC, lung cancer.

Previous study indicated that clonal mutations in ctDNA may provide more prognostic information than subclonal mutations in resectable non‐small cell lung cancer (NSCLC) [22]. This implies that the choice of tracked mutation types in a personalized panel may have a critical impact on the performance of MRD monitoring. To validate this, we compared the results of stratifying patients for recurrence prediction using clonal and subclonal mutations. Tumor purity estimates and tumor cellularity for each patient are provided in Table S5. The number of clonal and subclonal mutations in each patient's personalized panel, as well as the number of clonal and subclonal mutations detected in the ctDNA‐positive postoperative plasma samples, are shown in Figure 6A. Of note, clonal mutations were more frequently detected in MRD‐positive patients compared to subclonal mutations in CRC (93.3% vs. 70.4%), BC (88.9% vs. 71.4%), and LC (100% vs. 40%) patients (Figure 6A). The VAFs of the ctDNA clonal and subclonal mutations are shown in Figure S15. During postoperative MRD monitoring, we found that both clonal and subclonal mutations had prognostic value. Patients with detectable clonal ctDNA mutation had significantly worse PFS compared to those with negative detection of clonal ctDNA mutation (Figure 6B, HR = 7.43 with 95% CI: 2.58–21.44 for CRC; HR = 51.56 with 95% CI: 6.05–439.44 for BC; HR = 25.91 with 95% CI: 3.1–216.82 for LC; all *p* < 0.001). Similar results were observed when monitoring MRD using subclonal ctDNA (Figure 6B, *p* < 0.001, HR = 2.4 with 95% CI: 1.17–4.92 for CRC; *p* < 0.001, HR = 5.304 with 95% CI: 1.52–18.47 for BC; *p* = 0.066, HR = 6.301 with 95% CI: 1.14–34.76 for LC). However, using clonal mutations for patient risk stratification showed better predictive ability for cancer recurrence than using subclonal mutations. This holds true across CRC, BC, and LC, as illustrated in Figure 6B. These findings underscore the theoretical rationale for the preferential incorporation of clonal mutations into the personalized panel of our ctDNA‐MRD assay.

## 3 Discussion

Previous efforts in monitoring MRD have largely relied on either fixed panels or bespoke panels. In this study, we have developed the ctDNA‐MRD assay that integrates the advantages of both personalized and fixed panels. The integrated strategy offers three key advantages for MRD monitoring. First, it significantly improves probe capture efficiency and increases usable sequencing data yield compared to standalone personalized panels while maintaining experimental stability. Second, the fixed panel component serves as an internal quality control to identify and troubleshoot technical issues when assay anomalies occur. Most importantly, this approach not only allows us to perform the highly sensitive tracking of patient‐specific tumor mutations but also enables us to monitor certain therapeutic targets and high‐frequency mutations that may not be detected in tissue samples due to heterogeneity, or emerging mutations that may arise during tumor evolution or treatment, thereby the ctDNA‐MRD assay can accurately predict the recurrence risk for cancer patients.

This ctDNA‐MRD assay was developed based on carefully optimized DNA input and sequencing depth considerations. In this study, we determined a starting DNA input of 30 ng as the minimum requirement, while 80 ng as the maximum DNA input. While increasing DNA input can improve detection sensitivity, this approach is often limited by the available plasma volume, particularly in anemic cancer patients. We also observed that the effective sequencing depth reached saturation when DNA input exceeded 80 ng. Moreover, increasing DNA input cannot overcome an assay's fundamental background error rate limitations [4]. Therefore, to ensure accurate monitoring of low‐frequency variants, we have incorporated various strategies to reduce false positives in our assay. Leveraging unique identifiers (UIDs) allow for read clustering and error correction in both forward and reverse strands. We also established a comprehensive internal database for the control of background noise and filter of clonal hematopoiesis (CH) variants. It is well known that cancer‐derived cfDNA fragments tend to be shorter than those from normal cells. This information can also be utilized to filter out false positive mutations. Although deeper sequencing depth can also enhance ctDNA assay performance, we observed diminishing returns beyond 80,000× coverage, where duplication rates exceeded 80%. This indicates further increases in depth no longer significantly improve detection sensitivity but raise costs.

With our ctDNA‐MRD assay, we can achieve an LoD of 0.05% VAF and 0.1% VAF at single variant level using 60 and 30 ng of cfDNA as starting material. At the sample level, when monitoring two mutations, the LoD can reach 0.02% (60 ng) and 0.05% VAF (30 ng). When monitoring 12 or more mutations, the LoD can achieve as low as 0.005% (60 ng) at a high sequencing depth of ≧80,000×. By evaluating 660 negative loci from commercially available standards and 2200 negative loci from samples of healthy individuals, the negative percent agreement for variant detection was determined to be no less than 99.8%, thus demonstrating a LoB of 0% at 99.5% specificity. The high precision was confirmed by achieving 100% concordance in intra‐run (repeatability) and inter‐run (reproducibility) detection. We also observed a strong correlation between the measured ctDNA fractions and the expected ones, indicating the high accuracy of our assay in quantifying ctDNA content. In addition, exogenous and endogenous interferents, such as hemoglobin, isopropanol, and ethanol, did not significantly affect the accuracy of the assay. These results underscore the superior technical performance of the ctDNA‐MRD assay.

Prior research has established that the sensitivity of MRD monitoring increases with a higher number of loci monitored in personalized MRD assay [12]. However, this may be accompanied by a potential trade‐off in specificity [12], as well as an escalation in the costs. Hence, the selection of an optimal number of tracked mutations becomes paramount. In our ctDNA‐MRD assay, we typically monitor a range of 2–20 patient‐specific variants, with the following key considerations. First, by monitoring as few as two mutations, we can achieve a LoD at the sample level as low as 0.02%, which effectively meets the requirements of the majority of MRD monitoring scenarios. Second, monitoring more than 20 mutations may lead to a decrease in the specificity of MRD detection. Last but not least, attempting to monitor an excessive number of mutations would not only entail a lengthier personalized panel design cycle but also entail a substantial increase in sequencing costs. By meticulously determining the number of mutations to be tracked, we strike an optimal equilibrium between sensitivity, specificity, and cost‐effectiveness in MRD monitoring. Indeed, apart from the number, the type of tracked mutations is also crucial for MRD monitoring. We validated that clonal mutations have superior prognostic value compared to subclonal mutations. Therefore, we prioritize the inclusion of clonal mutations when selecting variants to be monitored. The refined design and implementation of our ctDNA‐MRD assay contribute to its clinical applicability and augur its potential for widespread integration into the realm of precision oncology. Beyond these advantages, our ctDNA‐MRD assay demonstrates significant cost‐effectiveness. Under comparable conditions, the experimental and analytical costs of our CGP panel are approximately one‐third those of WES for tumor tissue analysis. For ctDNA detection, the per‐sample cost of our assay represents a threefold reduction compared to conventional fixed panels.

Through postoperative MRD monitoring of a total of 89 solid tumor patients (46 CRC, 25 BC, and 18 LC), we observed high sensitivity (CRC: 93.8%; BC: 90%; LC: 88.9%) and specificity (CRC, BC, and LC: 100%) in predicting cancer recurrence. Our assay demonstrated a median lead time of 112 days for BC and 83 days for LC before imaging‐confirmed recurrence. This diagnostic lead time could enable the timely initiation of adjuvant or consolidation therapies, potentially improving patient outcomes. For example, Moding et al. showed that consolidation immunotherapy in NSCLC patients with post‐chemoradiation MRD significantly improved outcomes compared to untreated MRD‐positive patients, with early ctDNA dynamics further predicting therapeutic response [6]. Supporting this paradigm, ongoing clinical trials such as the ALTAIR trial (NCT04457297) evaluating preemptive trifluridine/tipiracil in MRD‐positive CLC and the Apollo study (NCT04501523) investigating escalated adjuvant immunotherapy for MRD‐positive triple‐negative BC underscore the translational potential of MRD‐guided adaptive treatment strategies. Although the current evidence for MRD‐guided adjuvant therapy is still limited, several studies have suggested that detectable MRD after curative treatment may indicate potential benefit from adjuvant therapy [6, 7, 20]. The cancer‐specific fixed panel in our ctDNA‐MRD assay includes common actionable variants for specific cancer types, enabling the proper selection of targeted therapy. Conversely, NSCLC patients with sustained undetectable MRD during long‐term postoperative monitoring may represent a potentially cured population, as they are less likely to benefit from adjuvant therapy or response to more aggressive and escalated treatment [6, 7]. Furthermore, a ctDNA‐guided approach to the treatment of stage II colon cancer has been demonstrated to reduce adjuvant chemotherapy without compromising progression‐free survival [23].

In the tracked variants, we found that clonal mutations demonstrated superior prognostic value compared to subclonal variants. The potential biological mechanisms underlying this observation may include the fact that clonal mutations are present in all cancer cells, whereas subclonal mutations are only present in a subset of tumor cells [24]. Clonal mutations can drive the initial steps in tumor development, and subsequent subclonal mutations can lead to further diversification within the tumor. It has been shown that the mutations of many known driver genes that have been recognized as prognostic markers or therapeutic targets were almost clonal, such as TP53, EGFR, and KRAS [24]. Moreover, in the context of tumor recurrence, clonal mutations, being present in all cancer cells, theoretically have a higher probability of detection compared to subclonal mutations. Most importantly, monitoring clonal mutations has been suggested to be a more sensitive ctDNA detection method than monitoring subclonal mutations. The TRACERx study [12], which involved ctDNA monitoring in 96 early‐stage NSCLC patients, found that 46 patients were preoperatively ctDNA‐positive. The authors demonstrated that clonal mutations were more frequently detected in ctDNA‐positive patients compared to subclonal mutations. All 46 ctDNA‐positive patients had detectable clonal mutations, with a median of 94% of the assay panel‐targeted clonal mutations detected in ctDNA (range, 11%–100%). In contrast, only 27 (68%, 27/40) patients had detectable subclonal mutations, with a median of 27% of the assay panel‐targeted subclonal mutations detected in ctDNA (range, 0%–91%). The average VAF of clonal mutations was also higher than that of subclonal mutations. These results collectively suggest that clonal mutations may have superior prognostic value compared to subclonal mutations. Therefore, we prioritize the inclusion of clonal mutations when selecting variants to be monitored.

Our study has several limitations. The tumor‐informed approach relies on the availability of tumor tissue, and the cohort's small sample size and limited cancer types constrain the validation of our ctDNA‐MRD assay's clinical utility. Further validation in larger, more diverse clinical cohorts is warranted. Given the retrospective design of our clinical validation cohort for CLC, with blood samples collected only at the time of recurrence for patients experiencing relapse, it is not possible to ascertain the lead time of the ctDNA‐MRD assay in comparison to imaging modalities for early recurrence detection of CLC. However, in the cohorts of BC and LC, this issue does not arise. Future prospective interventional studies should evaluate ctDNA‐MRD‐guided adaptive therapy strategies.

## 4 Conclusion

In conclusion, the ctDNA‐MRD assay with the integration of personalized panels and cancer‐specific fixed panels has demonstrated excellent analytical performance at both variant and sample levels. The ability of this assay to predict cancer recurrence based on ctDNA‐MRD monitoring also suggests exceptional clinical utility, characterized by high sensitivity and specificity. Our findings support the continued exploration and validation of the ctDNA‐MRD assay in larger clinical cohorts and broader cancer types, along with ongoing technical optimization, to ultimately enhance precision oncology practice and patient outcomes. Additionally, MRD‐based adaptive or intermittent treatment strategies also represent one of the most important directions for future research.

## 5 Materials and Methods

### 5.1 Patients and Samples

A total of 46 patients with CRC, 25 patients with triple‐negative BC, and 18 patients with NSCLC treated with definitive surgery were recruited for this study. Written informed consent was obtained from all participants. All patients provided tumor tissue formalin‐fixed paraffin‐embedded (FFPE) samples at the time of surgery. Peripheral blood samples (20 mL) were collected from all patients every 3–6 months after surgery for MRD monitoring. However, for CRC patients with recurrence, blood samples were only provided at the time of cancer recurrence. The clinical characteristics of CRC, BC, and LC patients were summarized separately in Tables S2–S4. Clinical recurrence was confirmed by radiographic imaging. A patient was considered MRD positive if ctDNA was detected at any time point during monitoring. Additionally, blood samples from 22 healthy donors were collected for analytical validation experiments.

### 5.2 Comprehensive Genomic Profiling of Tissue Samples

For tumor tissue samples, the FirePure FFPE gDNA Extraction Kit (FireGen, Beijing, China) was used to extract genomic DNA (gDNA). DNA concentration was measured using a Qubit fluorometer with the Qubit dsDNA High Sensitivity (HS) Assay Kit (Invitrogen, Carlsbad, CA, USA). The DNA fragment length was assessed using the LabChip NGS 3K Reagent Kit (Revvity Health Sciences, Waltham, MA, USA). Only gDNA samples above 200 ng and fragments within 200–250 bp were selected for further processing and analysis. Sequencing libraries were prepared using the DNBSEQ‐T7RS High‐throughput Sequencing Set (MGI Tech, Shenzhen, China) according to the manufacturer's instructions. A previously reported fixed panel [7, 8, 25] covering ∼2.3 Mbp of human genome and targeting 1021 cancer‐associated genes was utilized for CGP of tumor tissues. This panel was designed using a concept similar to CAPP‐seq [11]. Gene selection prioritized the most common driver mutations across solid tumors, followed by actionable alterations linked to treatment sensitivity, resistance, and immunotherapy response. Finally, frequently mutated regions were incorporated based on data from 48,353 Geneplus‐sequenced cancer patients and public databases, such as Catalogue Of Somatic Mutations In Cancer (COSMIC) and The Cancer Genome Atlas (TCGA).

### 5.3 Tumor Somatic Variant Calling

The sequenced reads were mapped to the reference human genome (GRCh37) using the default parameters in BWA‐mem2 version 2.2.1 after removing adaptor and low‐quality reads. Duplicated reads were marked and removed using realSeq2 (version 3.0.6; Geneplus‐Beijing, in‐house) for tumor and germline genomic DNA. Tumor somatic SNVs and small InDels were profiled using realDcaller2 (v2.0.10; Geneplus‐Beijing, in‐house) and Mcaller (v0.0.3 Geneplus‐Beijing, in‐house). We developed a decision tree model to discriminate between germline and somatic mutations. The model incorporated three features: mutation frequency (caseAF), which represents the detected mutation frequency in the analyzed sample; mutation count in the PAD database (PAD_count), indicating the recurrence of the mutation in the germline baseline database (PAD), which was established with 89,767 cancer patients who underwent genetic testing in GenePlus Co., Ltd and includes mutations identified in white blood cell samples; and the *p*‐value obtained from the two‐tailed binomial test comparing the observed minor allele frequency (MAF) of variants with their theoretical MAF. The single‐nucleotide polymorphisms with >1% population allele frequency in ExAc or 1000 Genomes Project were filtered, and the variant positional depth was at least >50X. Furthermore, we have developed a baseline population database (POT) containing 2000 samples without tumor mutations, which serves as a reference for filtering out background noise. Detailed descriptions can be found in our previous study [26].

### 5.4 The Algorithm for Selecting Patient‐Specific Somatic Variants

In our study, patient‐specific monitoring variant selection and probe sequence design were conducted using the GenePlus platform's proprietary algorithm, with the biotinylated capture probes synthesized in‐house under standardized protocols. Our tumor tissue‐informed MRD target selection process prioritized oncogenic driver mutations and clonal variants exhibiting the highest variant allele frequencies (VAFs), selecting a maximum of 20 top‐ranked variants per patient. Clonal mutations were identified using PycloneVI [27] with the following parameters: “–num‐annealing‐steps 1,” “–num‐clusters 10,” “–density binomial,” “–num‐grid‐points 100,” “–num‐restarts 1,” “–annealing‐power 1,” “–convergence‐threshold 1.00E‐06,” “–max‐iters 10000,” “–mix‐weight‐prior 1,” “–precision 200,” “–seed None.” The selection process incorporated several technical filters to ensure optimal performance. Variants located in repetitive elements, short tandem repeat regions, high‐GC content regions (>80%), or areas with significant sequence homology (>90% identity) were excluded. Variants with elevated background noise rates were filtered against an in‐house baseline database constructed from >3000 healthy donor samples.

Probe design was customized according to variant type. For SNVs, probes were selected based on optimal variant locus positioning, balanced GC content, and minimal sequence homology. For indels, we employed tri‐probe sets consisting of one mutant‐specific probe and two flanking reference probes. For SVs, breakpoint‐spanning fusion probes were designed along with partner gene probes.

### 5.5 Tumor‐Naive Cancer‐Type‐Specific Panel Design

We have developed cancer‐specific panels for LC, CLC, BC, and pan‐cancer. These panels include class I actionable mutations, which are FDA‐approved drug targets, or recommended by consensus guidelines, or clinically validated resistance‐related mutations. We also incorporated hotspot mutation, driver mutation, and high‐frequency mutation from publicly available databases such as TCGA and COSMIC for specific cancer types. Additionally, 21 SNP probes from the dbSNP database [28] were included, targeting SNPs with high heterozygosity to determine the sample source of tumor and blood samples, enabling us to discern samples derived from the same patient. The cancer‐type‐specific panel (∼5 kb), along with personalized panel, will be incorporated to be used for ctDNA‐MRD monitoring in cancer patients.

### 5.6 Plasma cfDNA Library Preparation and Sequencing

Peripheral blood samples were collected using Streck tubes (Streck Corporate, Omaha, NE, USA) and processed within 72 h after collection. Blood samples were centrifuged at 1600 × *g* for 10 min, followed by a subsequent centrifugation at 16,000 × *g* for 10 min to remove cellular debris. For each blood sample, 3–8 mL (median 8 mL) of plasma was used for cfDNA extraction using MagMAX Cell‑Free DNA Isolation Kit (Thermo Fisher, Carlsbad, CA, USA). The concentration and fragment length of cfDNA were assessed using the same methods as tissue DNA mentioned above. Only cfDNA samples greater than 30 ng and exhibiting fragments of approximately 170 bp were selected for further processing and analysis. For samples with cfDNA quantities ranging from 30 to 80 ng, all cfDNA was used for library preparation. However, for samples with DNA quantities more than 80 ng, a maximum of 80 ng cfDNA was utilized. For library preparation, cfDNA was pre‐processed by end‐repairing and A‐tailing reactions. Sequencing adapters with UIDs were ligated to the ends of cfDNA fragments, and PCR was performed to generate sufficient amounts of fragments prior to hybridization. These libraries were then hybridized to custom‐designed probes covering the sequences of target regions. Subsequently, DNA sequencing was performed using a DNBSEQ‐T7 (MGI Tech, Shenzhen, China) or Gene+Seq‐2000 sequencer at PE100 read‐length with an ultra‐deep sequencing depth of ≧80,000× (100,000× in ∼98% samples).

### 5.7 cfDNA Sequencing Data Processing

Raw reads were processed to remove adapter sequences and low‐quality reads. The remaining reads were aligned to the reference human genome (GRCh37) using the default parameters in BWA‐mem2 version 2.2.1. Duplicated reads were identified by UIDs and the position of template fragments to eliminate errors introduced by PCR or sequencing using realSeq2 (version 3.0.6; Geneplus‐Beijing, in‐house). Quality control assessment was performed using abra2 (version 2.24‐0.5; Broad Institute).

### 5.8 ctDNA‐MRD Detection

After correcting sequencing errors using UID, SNV calling was performed using a custom bioinformatics pipeline optimized for detecting ultra‐low‐frequency mutations. SNV and InDel calling was carried out mainly by realDcaller2 (v2.0.10; Geneplus‐Beijing, in‐house), while Mcaller (v0.0.3 Geneplus‐Beijing, in‐house) was used as auxiliary software to improve the detection of long InDels.

Then, the following strategies were integrated into bioinformatic workflows eliminating the background noise to ensure the specificity of the detection of PSVs in ctDNA. For each PSV, a background noise model was constructed by the double‐strand consensus (DS) sequence reads detected at PSV's genome position. Here, the assumption is that if one strand of the DS sequence reads supports the wild allele, the other strand should also support it, since they originate from the same DNA molecule. Therefore, based on this principle, the background noise of a specific mutant allele is calculated as the frequency of the mutant allele signal observed in one strand of the DS sequence reads, under the condition that the other strand supports the wild allele. Then the assumption of a Poisson distribution was used to calculate the significance of each PSV. For a given PSV with an observed coverage depth of *n* and the corresponding background noise of e, when the number of PSV's mutant reads is n, the probability (*p* value) of detecting this PSV by chance is:

$$P_{\mathrm{mut}}=1-\sum_{k\;=0}^{n-1}\frac{\lambda^{k}}{k!}e^{-\lambda }$$

The PSV was determined as positive if $P_{\mathrm{mut}}$ < 0.05.

A two‐step strategy was used to determine the MRD status of the plasma sample. First, the ctDNA fraction in plasma samples was estimated based on all PSVs using the maximum likelihood (ML) method. Then, the null hypothesis, a negative MRD status, was tested by the likelihood ratio test to determine the MRD status of plasma sample.

In detail, assuming that the ctDNA fraction is $tf_{p}$, the VAF of the ith PSV in the tumor tissue sample is $\mathrm{ta}f_{i}$, and the corresponding background noise in the plasma sample is $e_{i}$, then the expectation of VAF (${\mathrm{af}}_{\mathrm{theory}}^{i}$) in the plasma sample satisfies:

$${\mathrm{af}}_{\mathrm{theory}}^{i}\left(tf_{p}\right)=\frac{\mathrm{ta}f_{i}\cdot tf_{p}}{2\cdot \mathrm{tmsaf}}+\left(1-\frac{\mathrm{ta}f_{i}\cdot tf_{p}}{2\cdot \mathrm{tmsaf}}\right)\cdot e_{i}$$

where tmsaf is the max VAF of somatic variants detected in tumor tissue. The assumption of a Poisson distribution was used to model the detected $n_{\mathrm{alt}}^{i}$ mutant reads of the ith PSV, with an observed coverage depth of $n_{\mathrm{total}}^{i}$:

$$n_{\mathrm{alt}}^{i}∼\mathrm{poisson}\left(\lambda^{i}\left(tf_{p}\right)\right)$$

where $\lambda^{i}\;\left(tf_{p}\right)=n_{\mathrm{total}}^{i}\cdot {\mathrm{af}}_{\mathrm{theroy}}^{i}\left(tf_{p}\right)$. The posterior probability of the ith PSV and the equation is as follows:

$$p^{i}\;\left(n_{\mathrm{alt}}^{i}=n^{i}\;|tf_{p}\right)=\;\mathrm{poisson}\left(n^{i},\lambda^{i}\left(tf_{p}\right)\right)$$

where $n^{i}$ represents the detected number of mutant reads of the ith PSV in the plasma sample. The log‐likelihood function of $tf_{p}$ is:

$$l\left(tf_{p}\right)=\prod_{i=1}^{N}p^{i}\left(n_{\mathrm{alt}}^{i}=n^{i}|tf_{p}\right)=\prod_{i=1}^{N}\mathrm{poisson}\left(n^{i},\lambda^{i}\left(tf_{p}\right)\right)$$

The ctDNA fraction $\hat{tf_{p}}$ was obtained by performing ML estimation on $tf_{p}$:

$$\hat{tf_{p}}=\arg_{tf_{p}}^{\max}\;\prod_{i=1}^{N}p^{i}\left(\;n_{\mathrm{alt}}^{i}=n^{i}\;|tf_{p}\right)=\arg_{tf_{p}}^{\max}\;\prod_{i=1}^{N}\mathrm{poisson}\left(n^{i},\lambda^{i}\left(tf_{p}\right)\right)$$

$$=\arg_{tf_{p}}^{\max}\;\ln\left(\prod_{i=1}^{N}\mathrm{poisson}\left(n^{i},\lambda^{i}\left(tf_{p}\right)\right)\right)$$

$$=\arg_{tf_{p}}^{\max}\;\sum_{i=1}^{N}\ln\left(\mathrm{poisson}\left(n^{i},\lambda^{i}\left(tf_{p}\right)\right)\right)$$

The null hypothesis $tf_{p}=0$ was tested by the likelihood ratio test, and the likelihood ratio statistic is as follows:

$$\mathrm{LRT}\;\left(\hat{tf_{p}}\right)=2\left(L\left(0\right)-L\left(\hat{tf_{p}}\right)\right)∼\chi_{1}^{2}$$

The *p* value was then calculated using the probability density function of the $\chi_{1}^{2}$ distribution:

$$P_{\mathrm{sample}}=P\left(x\geq \mathrm{LRT}\left(\hat{tf_{p}}\right)\right)$$

The plasma sample was determined as positive if $P_{\mathrm{sample}}$ < 0.05.

### 5.9 Estimation of MTM

The MTM per milliliter of plasma was calculated using ctDNA fraction, along with the cfDNA mass (ng) and plasma volume (mL) used for cfDNA extraction, as follows:

$$\;\mathrm{MTM}/\mathrm{mL}=\frac{\mathrm{ctDNA}\;\mathrm{fraction}\;\times \;\mathrm{cfDNA}\;\mathrm{mass}\;\left(\mathrm{ng}\right)\times 1000\;\mathrm{pg}/\mathrm{ng}\;}{3.3\;\mathrm{pg}\;\times \;\mathrm{plasma}\;\mathrm{volume}\;\left(\mathrm{mL}\right)}$$

### 5.10 Theoretical Estimation for the Limit of Detection

*F*(*x*) equals the detection probability for positive mutations with mutation frequency *p* under the conditions of effective sequencing depth *n* and the positive predictive value *x*.

$$F\left(x\right)=P\left(X\geq x\right)=\sum_{k=x}^{n}\frac{n!}{k!(n-k)!}p^{k}{\left(1-p\right)}^{n-k}$$

When *F*(*x*) ≥ 95%, the minimum mutation frequency *p* is regarded as the LoD value.

### 5.11 Analytical Performance Validation

We conducted comprehensive analytical validation experiments to evaluate the performance of our personalized MRD assay. Its LoD at both the site level and sample level, and precision and stability were assessed using a commercially available MRD standard sample (GeneWell, GW‐OCTM800). We analyzed 21 SNVs, seven InDels, and five SVs in this standard sample. All mutations are listed in Table S1. These mutations are distributed across five gradient levels, with allele frequencies of 0.1%, 0.05%, 0.02%, 0.01%, and 0.005%, respectively. We also evaluated the LoB of our assay using the mutations with 0% VAF in the standard sample and cfDNA samples from healthy individuals. To verify the accuracy of ctDNA quantification, we prepared a SW480 cell line DNA standard sample. The SW480 DNA was fragmented and mixed with normal DNA from the NA12878 cell line to generate samples with a ctDNA fraction of 0.1%. The accuracy of the dilution process was verified using droplet digital PCR (ddPCR). Subsequently, the 0.1% samples were diluted in a serial manner to ctDNA fractions of 0.05%, 0.02%, 0.01%, and 0.005%. For these dilution gradients falling below the detection limit of the ddPCR assay (0.1%), changes in the exogenously introduced viral DNA copies were employed as indicators of the dilution ratio.

### 5.12 Statistical Analysis

All statistical analyses were performed using R software (v4.3.1). Paired *t*‐tests were utilized for comparisons within paired samples, Mann–Whitney tests were employed to compare differences between two groups, and Fisher's exact test was utilized to analyze the association or significance between two categorical variables in our study. Pearson's correlation analysis was used to analyze the consistency of expected and detected VAFs in the standard sample. Kaplan–Meier plots were generated by R package survival (version 3.5.5) and survminer (version 0.4.9) to evaluate the progression‐free survival (PFS) based on the status of MRD (positive or negative). The log‐rank test was employed to compare the survival curves. Statistical significance was defined as a *p*‐value less than 0.05.

## Author Contributions

Jianxing He and Xin Yi conceived the projects and supervised the study. Liping Liu and WenHua Liang conducted data collection and analysis. HengRui Liang, Liyan Huang, Danman Zhong, Qiuhua Deng, Wei Wang, Jun Huang, Chao Yang, and GuiLin Peng contributed to patient enrollment and sample collection. WuQiang Cao, Wei Gao, Yan Zhang, XiaoLing Zeng, XunMei Zheng, JiaXin Ma, XinHua Du, and Yanfang Guan developed the ctDNA assay and bioinformatics analysis methods. Hongke Wang performed data visualization and drafted the manuscript. Wei Gao, XinHua Du, Yanfang Guan, and Xuefeng Xia provided insights into methodological approaches and analysis. WuQiang Cao, Wei Gao, Liang Cui, and Jing Bai proofread the manuscript. All authors have read and approved the final manuscript.

## Conflicts of Interest

WuQiang Cao, Hongke Wang, Wei Gao, Yan Zhang, XiaoLing Zeng, XunMei Zheng, JiaXin Ma, XinHua Du, Liang Cui, Yanfang Guan, Jing Bai, Xuefeng Xia, and Xin Yi are the employees in Geneplus but have no potential relevant financial or non‐financial interests to disclose. The other authors have no conflicts of interest to declare.

## Ethics Statement

This study was approved by the Medical Ethics Committee of The First Affiliated Hospital of Guangzhou Medical University [ES‐2024‐164‐01]. All the participants provided their written informed consent for this study.

## Supporting information




**Figure S1**: Patients covered by our cancer type specific fixed panels.
**Figure S2**: Analysis of pre‐analytical variables.
**Figure S3**: Detection of variants with 0.02%, 0.01%, and 0.005% VAFs.
**Figure S4**: The limit of detection for the ctDNA‐MRD assay at variant level.
**Figure S5**: The specificity analysis at variant and sample level.
**Figure S6**: Stability analysis of the ctDNA‐MRD assay.
**Figure S7**: Repeatability and reproducibility analysis of the ctDNA‐MRD assay.
**Figure S8**: Event chart depicting the follow‐up history of postoperative MRD testing and disease relapse in colorectal cancer (CRC) patients.
**Figure S9**: Summary of patient‐specific variants and tumor burden of colorectal cancer patients.
**Figure S10**: Summary of cfDNA concentration and sequencing depth.
**Figure S11**: Event chart depicting the follow‐up history of postoperative MRD testing and disease relapse in breast cancer (BC) patients.
**Figure S12**: Summary of patient‐specific variants and tumor burden of breast cancer patients.
**Figure S13**: Event chart depicting the follow‐up history of postoperative MRD testing and disease relapse in lung cancer (LC) patients.
**Figure S14**: Summary of patient‐specific variants and tumor burden of lung cancer patients.
**Figure S15**: The variant allele frequencies of the ctDNA clonal or subclonal mutations in postoperative MRD‐positive patients.
Supplementary Tables
**Table S1**: Mutation list of ctDNA standard sample.
**Table S2**: Clinical characteristics of 46 patients with colorectal cancer. Related to Figure 5.
**Table S3**: Clinical characteristics of 25 patients with triple negative breast cancer. Related to Figure 5.
**Table S4**: Clinical characteristics of 18 patients with lung cancer. Related to Figure 5.

Supporting File 2: mco270483‐sup‐0002‐TableS5.xlsx

## Data Availability

The raw DNA sequencing data are available from the corresponding authors upon formal and reasonable request.
