# Supplementary material for: Personalized and Tumor Informed Circulating Tumor DNA Assay for Molecular Residual Disease Monitoring of Solid Malignancies
Source: MedComm (2020). 2025 Dec 3;6(12):e70483. doi: 10.1002/mco2.70483 (PMC12674089; doi:10.1002/mco2.70483)
Supplement: Supplementary file 1 — Figure S1: Patients covered by our cancer type specific fixed panels. Figure S2: Analysis of pre‐analytical variables. Figure S3: Detection of variants with 0.02%, 0.01%, and 0.005% VAFs. Figure S4: The limit of detection for the ctDNA‐MRD assay at variant level. Figure S5: The specificity analysis at variant and sample level. Figure S6: Stability analysis of the ctDNA‐MRD assay. Figure S7: Repeatability and reproducibility analysis of the ctDNA‐MRD assay. Figure S8: Event chart depicting the follow‐up history of postoperative MRD testing and disease relapse in colorectal cancer (CRC) patients. Figure S9: Summary of patient‐specific variants and tumor burden of colorectal cancer patients. Figure S10: Summary of cfDNA concentration and sequencing depth. Figure S11: Event chart depicting the follow‐up history of postoperative MRD testing and disease relapse in breast cancer (BC) patients. Figure S12: Summary of patient‐specific variants and tumor burden of breast cancer patients. Figure S13: Event chart depicting the follow‐up history of postoperative MRD testing and disease relapse in lung cancer (LC) patients. Figure S14: Summary of patient‐specific variants and tumor burden of lung cancer patients. Figure S15: The variant allele frequencies of the ctDNA clonal or subclonal mutations in postoperative MRD‐positive patients. Supplementary Tables Table S1: Mutation list of ctDNA standard sample. Table S2: Clinical characteristics of 46 patients with colorectal cancer. Related to Figure 5. Table S3: Clinical characteristics of 25 patients with triple negative breast cancer. Related to Figure 5. Table S4: Clinical characteristics of 18 patients with lung cancer. Related to Figure 5. [file MCO2-6-e70483-s001.docx]

**Supporting Information**

[Supplementary Figures 2](#_Toc209196309)

[Fig. S1. Patients covered by our cancer type specific fixed panels. 2](#_Toc209196310)

[Fig. S2. Analysis of pre-analytical variables. 3](#_Toc209196311)

[Fig. S3. Detection of variants with 0.02%, 0.01%, and 0.005% VAFs. 4](#_Toc209196312)

[Fig. S4. The limit of detection for the ctDNA-MRD assay at variant level. 5](#_Toc209196313)

[Fig. S5. The specificity analysis at variant and sample level. 6](#_Toc209196314)

[Fig. S6. Stability analysis of the ctDNA-MRD assay. 7](#_Toc209196315)

[Fig. S7. Repeatability and reproducibility analysis of the ctDNA-MRD assay. 8](#_Toc209196316)

[Fig. S8. Event chart depicting the follow-up history of postoperative MRD testing and disease relapse in colorectal cancer (CRC) patients. 9](#_Toc209196317)

[Fig. S9. Summary of patient-specific variants and tumor burden of colorectal cancer patients. 10](#_Toc209196318)

[Fig. S10. Summary of cfDNA concentration and sequencing depth. 11](#_Toc209196319)

[Fig. S11. Event chart depicting the follow-up history of postoperative MRD testing and disease relapse in breast cancer (BC) patients. 12](#_Toc209196320)

[Fig. S12. Summary of patient-specific variants and tumor burden of breast cancer patients. 13](#_Toc209196321)

[Fig. S13. Event chart depicting the follow-up history of postoperative MRD testing and disease relapse in lung cancer (LC) patients. 14](#_Toc209196322)

[Fig. S14. Summary of patient-specific variants and tumor burden of lung cancer patients. 15](#_Toc209196323)

[Fig. S15. The variant allele frequencies of the ctDNA clonal or subclonal mutations in postoperative MRD-positive patients. 16](#_Toc209196324)

[Supplementary Tables 17](#_Toc209196325)

[Table S1. Mutation list of ctDNA standard sample. 17](#_Toc209196326)

[Table S2. Clinical characteristics of 46 patients with colorectal cancer. Related to Figure 5. 18](#_Toc209196327)

[Table S3. Clinical characteristics of 25 patients with triple negative breast cancer. Related to Figure 5. 19](#_Toc209196328)

[Table S4. Clinical characteristics of 18 patients with lung cancer. Related to Figure 5. 20](#_Toc209196329)

# Supplementary Figures


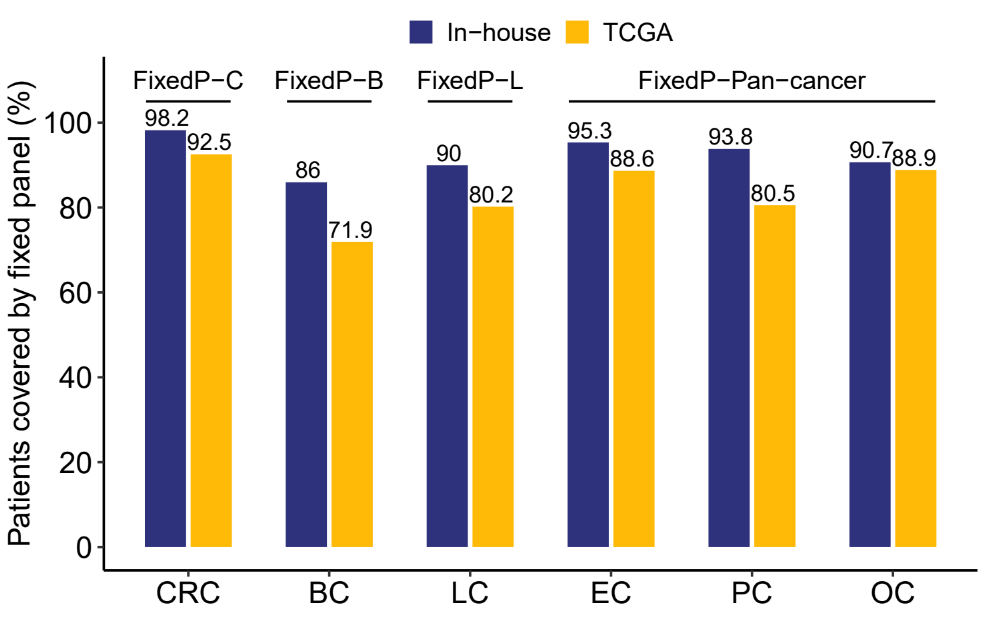


## Fig. S1. Patients covered by our cancer type specific fixed panels.

The bar chart displays the proportions of patients covered by our cancer-type-specific fixed panels in both the In-house data and TCGA database. Patients who have at least one mutation detected in the cancer-type-specific panel were considered covered. The In-house data includes 1178 CRC, 2060 LC,1068 BC, 321 EC, 580 PC, and 665 OC cases. And we totally used 522 CRC, 1005 LC, 994 BC, 185 EC, 185 PC, and 458 OC datasets from TCGA database. CRC: colorectal cancer, BC: breast cancer, LC: lung cancer, EC: esophageal cancer, PC: pancreatic cancer, OC: ovarian cancer; FixedP-C: fixed panel for CRC, FixedP-B: fixed panel for BC, FixedP-L: fixed panel for LC, FixedP-Pan-cancer: fixed panel for pan-cancer (EC, PC, OC, and other common solid tumors).


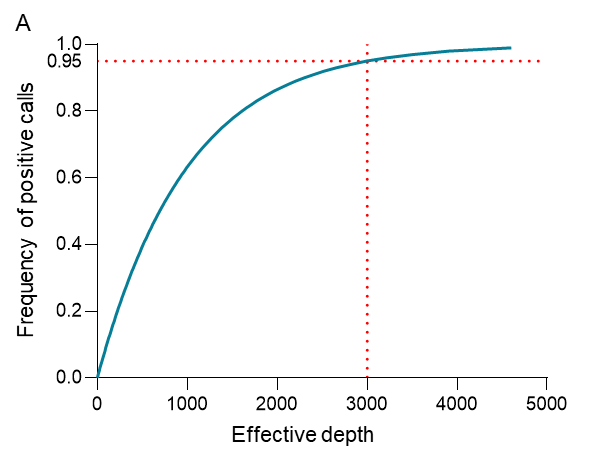


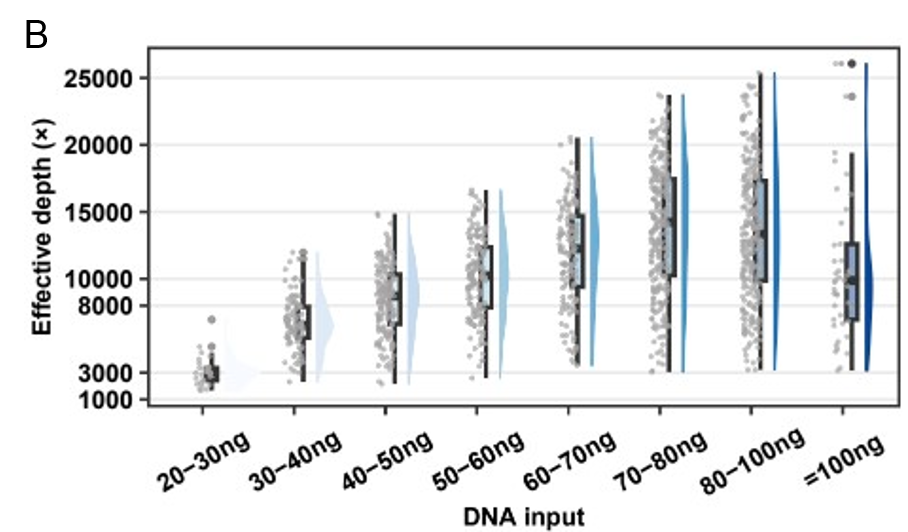


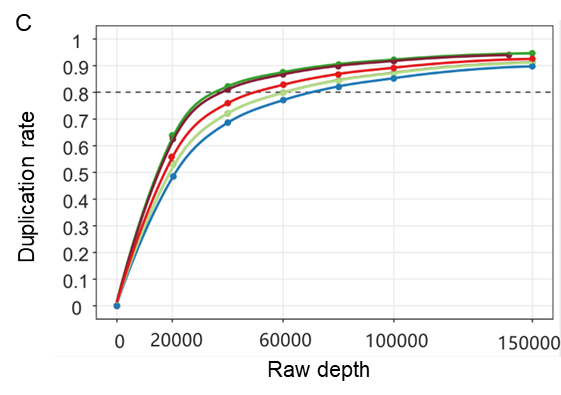


## Fig. S2. Analysis of pre-analytical variables.

**A**. The frequency of detecting at least one positive mutation under different effective sequencing depths, simulated using the binomial distribution. **B**. The effective sequencing depth under different DNA input. **C**. The duplication rate of reads obtained under different raw depth of sequencing reads. Each line represents a plasma ctDNA sample.


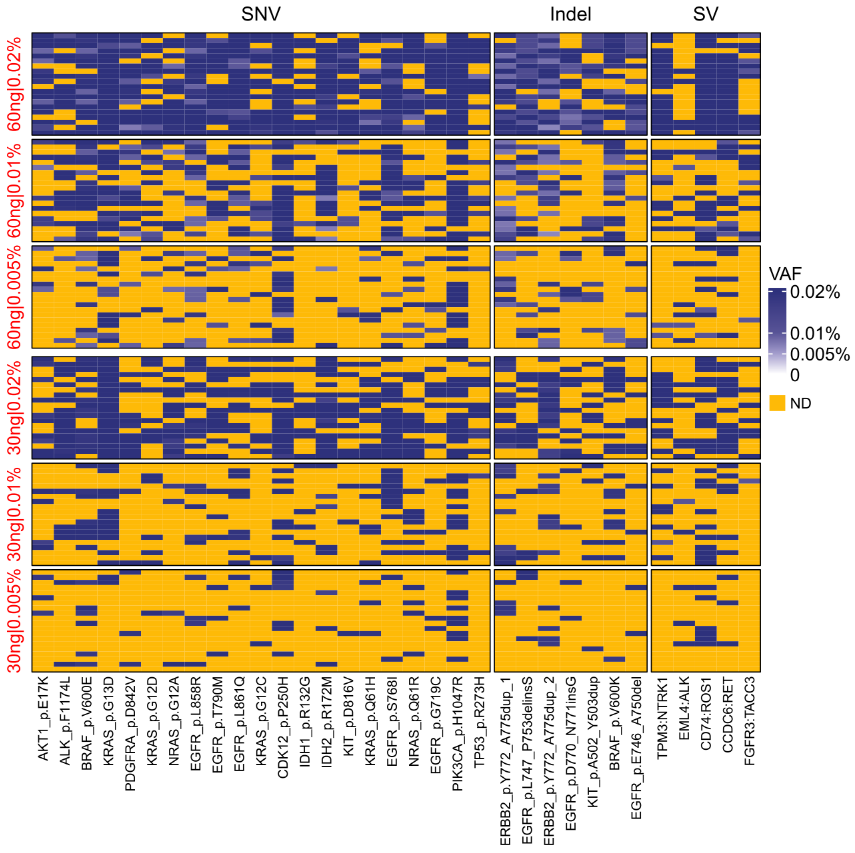


## Fig. S3. Detection of variants with 0.02%, 0.01%, and 0.005% VAFs.

At the DNA input of 60 ng and 30 ng, the detected mutations (dark blue) and missed mutations (gold) were shown in 20 samples carrying 33 mutations (21 SNVs, 7 InDels, and 5 SVs) at variant allele frequencies (VAFs) of 0.02%, 0.01%, and 0.005%. Each row represents a sample, while each column represents a monitored variant. ND: not detected.


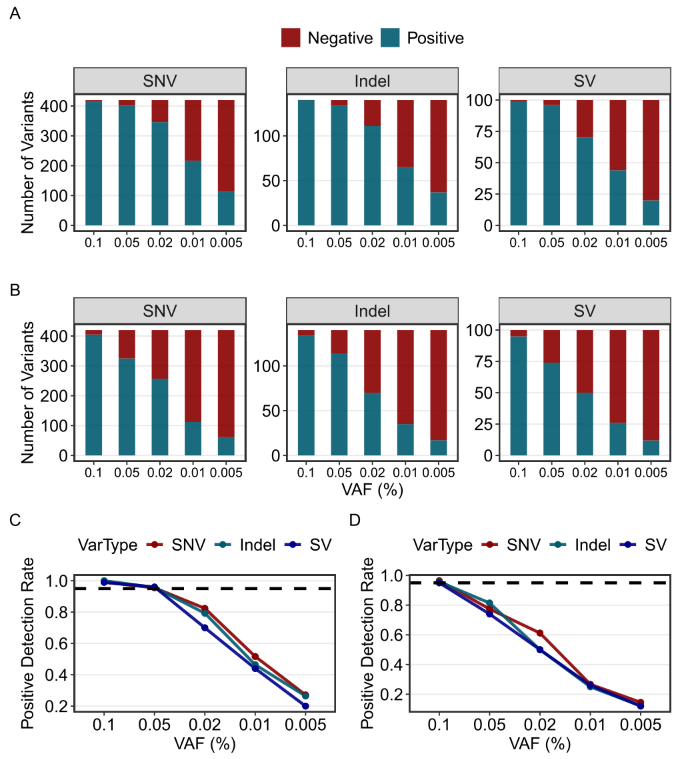


## Fig. S4. The limit of detection for the ctDNA-MRD assay at variant level.

At variant allele frequencies (VAFs) of 0.1%, 0.05%, 0.02%, 0.01%, and 0.005%, the number of detected mutations (positive) and missed mutations (negative) with the DNA input of 60ng (**A**) and 30ng (**B**), as well as the corresponding mutation detection rates with the DNA input of 60ng (**C**) and 30ng (**D**), were determined for SNVs, Indels, and SVs, separately. The black dashed line in the bottom panel denotes the 95% positive detection rate.


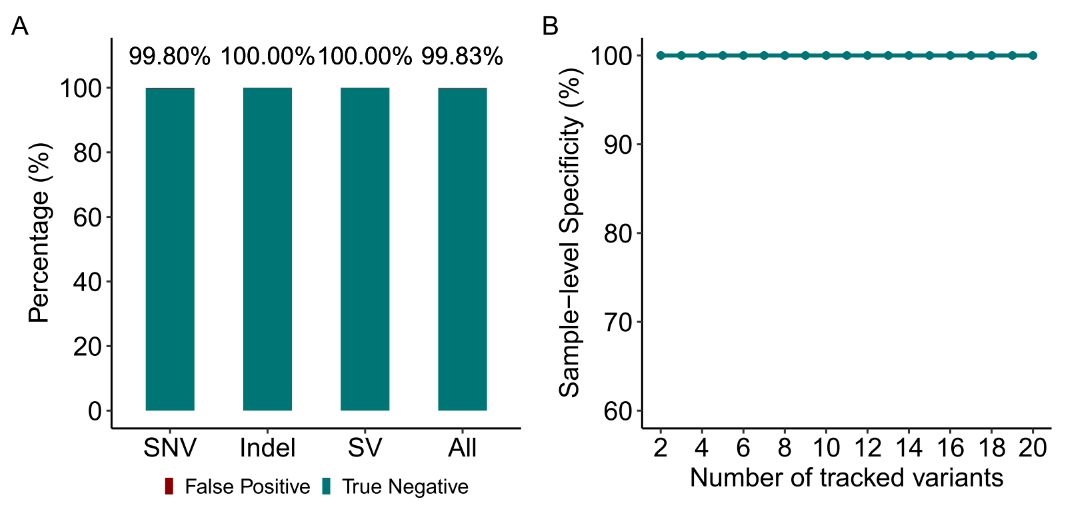


## Fig. S5. The specificity analysis at variant and sample level.

(**A**) For SNVs, Indels, and SVs, bar charts were presented to illustrate the proportions of true negatives (TN) and false positives (FP). And the variant-level specificity was annotated at the top of each bar plot. (**B**) The specificity of the 1021-MRD assay at the sample level for monitoring 2 to 20 mutations was displayed.


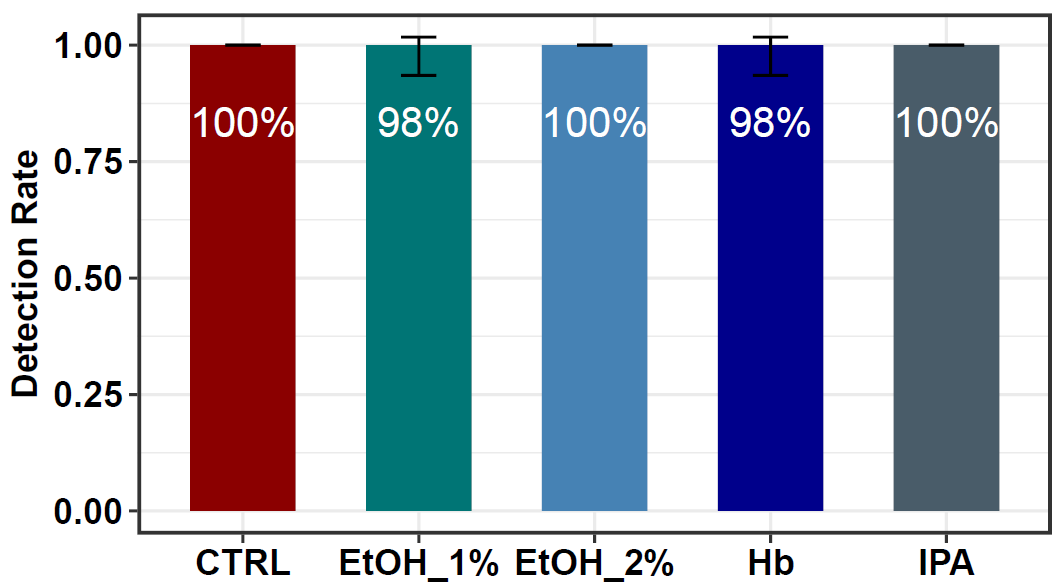


## Fig. S6. Stability analysis of the ctDNA-MRD assay.

The bar plot shows average mutation detection rates ± standard deviation in the positive standard samples of different experimental groups. Each group was repeated three times. CTRL: no interferent control, EtOH_1%: 1% ethanol, EtOH_2%: 2% ethanol, Hb: 2 mg/mL hemoglobin, IPA: 1% isopropanol.


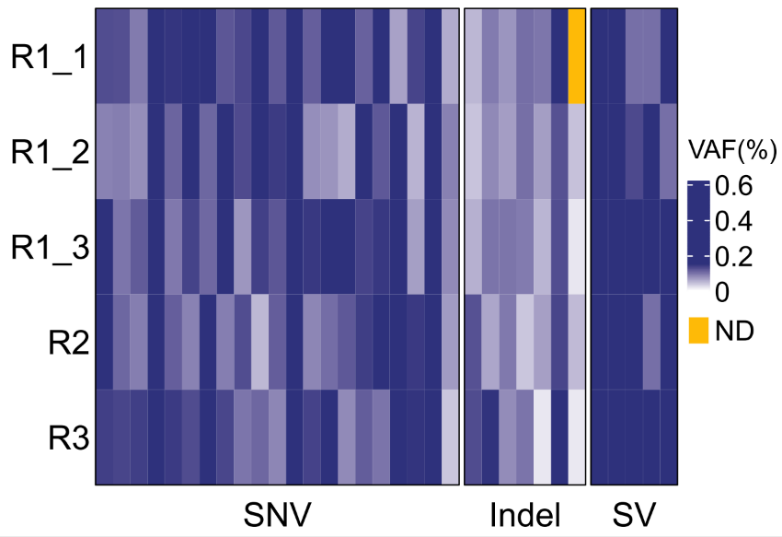


## Fig. S7. Repeatability and reproducibility analysis of the ctDNA-MRD assay.

The variant allele frequencies (VAFs) of all 33 variants obtained from the 5 tests were displayed. Among them, R1_1, R1_2, and R1_3 were performed by the same operator in a single experiment, while R2 and R3 were independently conducted by two other operators. The experiment utilized commercially available standard samples with 30ng DNA input. Missed mutation was marked with gold color. ND: not detected.


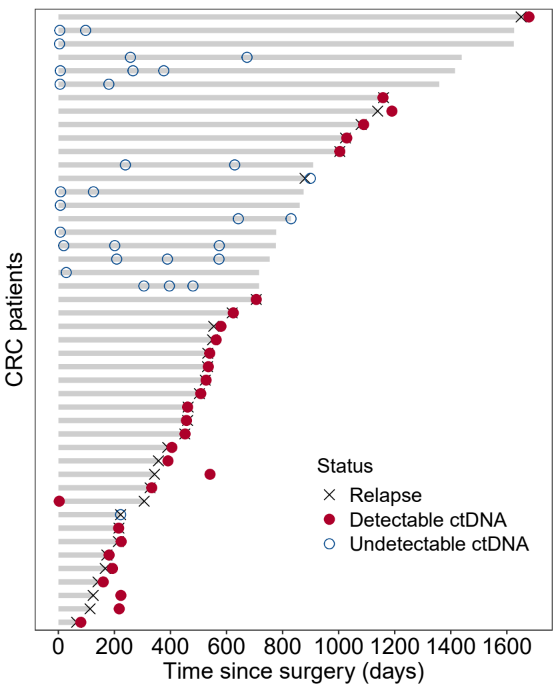


## Fig. S8. Event chart depicting the follow-up history of postoperative MRD testing and disease relapse in colorectal cancer (CRC) patients.


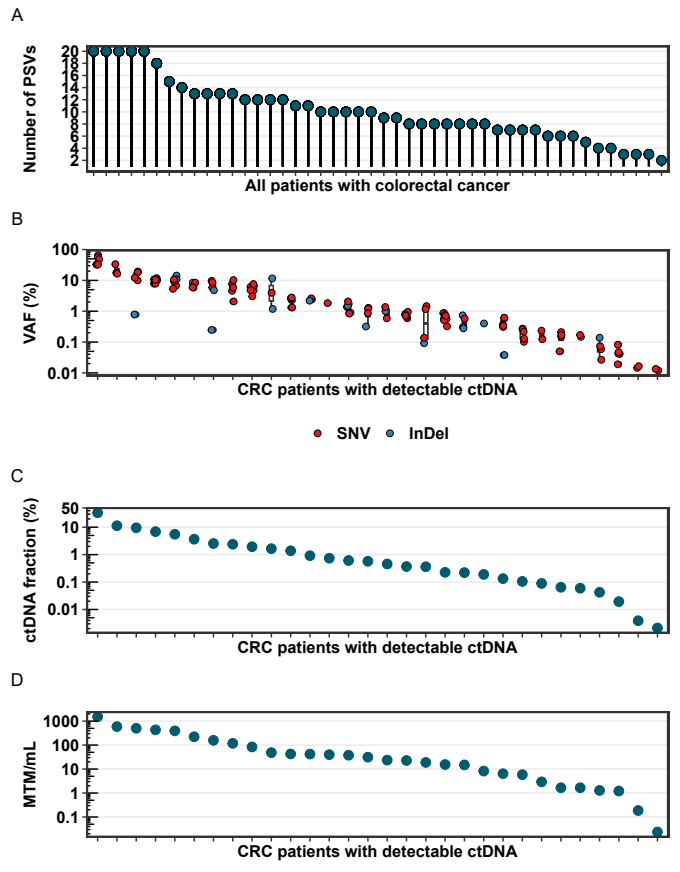


## Fig. S9. Summary of patient-specific variants and tumor burden of colorectal cancer patients.

(**A**) The number of patient-specific variants incorporated into the personalized panel for each CRC patient. (**B**) The ctDNA variant allele frequency (VAF), (**C**) ctDNA fraction and (**D**) mean tumor molecules (MTM/mL) in the postoperative plasma with detectable ctDNA of CRC patients. CRC: colorectal cancer.


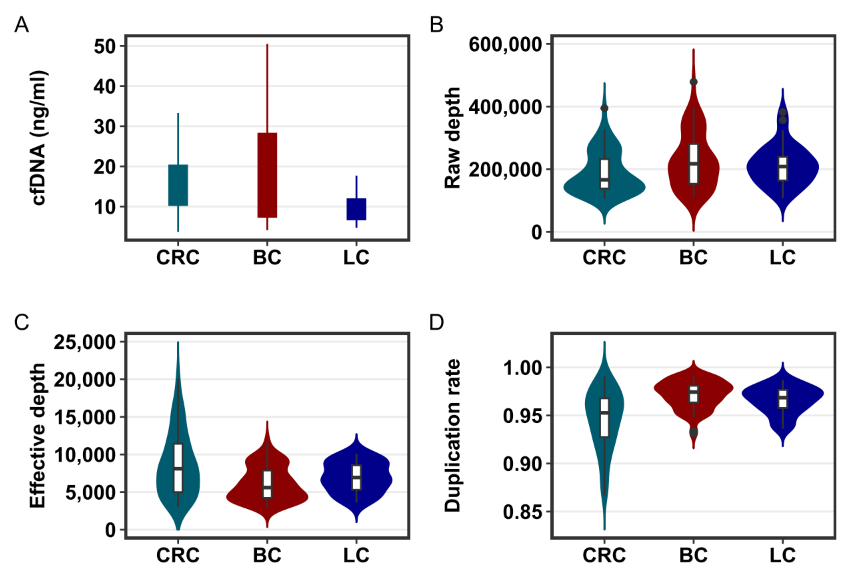


## Fig. S10. Summary of cfDNA concentration and sequencing depth.

The distribution of (**A**) cfDNA concentration (ng/mL), (**B**) raw depth, (**C**) effective depth, and (**D**) duplication rate of sequencing reads. CRC: colorectal cancer, BC: breast cancer, LC: lung cancer.


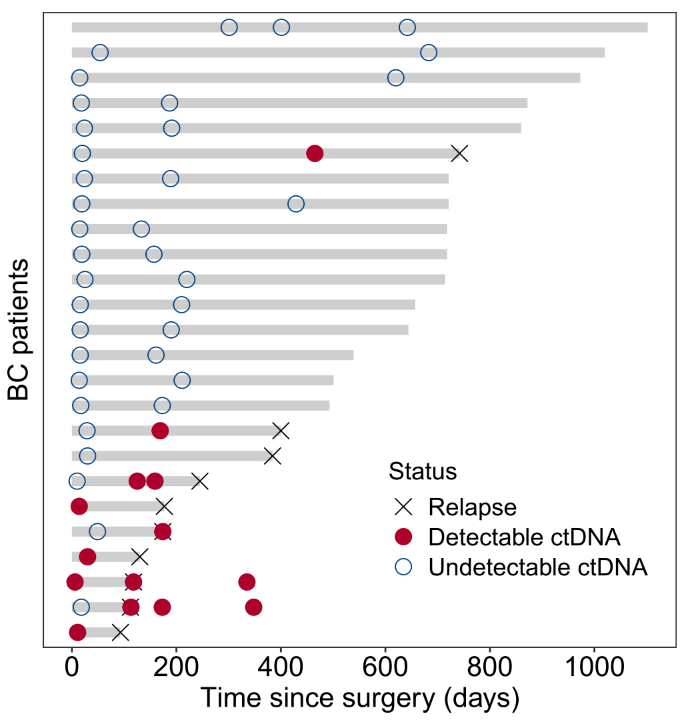


## Fig. S11. Event chart depicting the follow-up history of postoperative MRD testing and disease relapse in breast cancer (BC) patients.


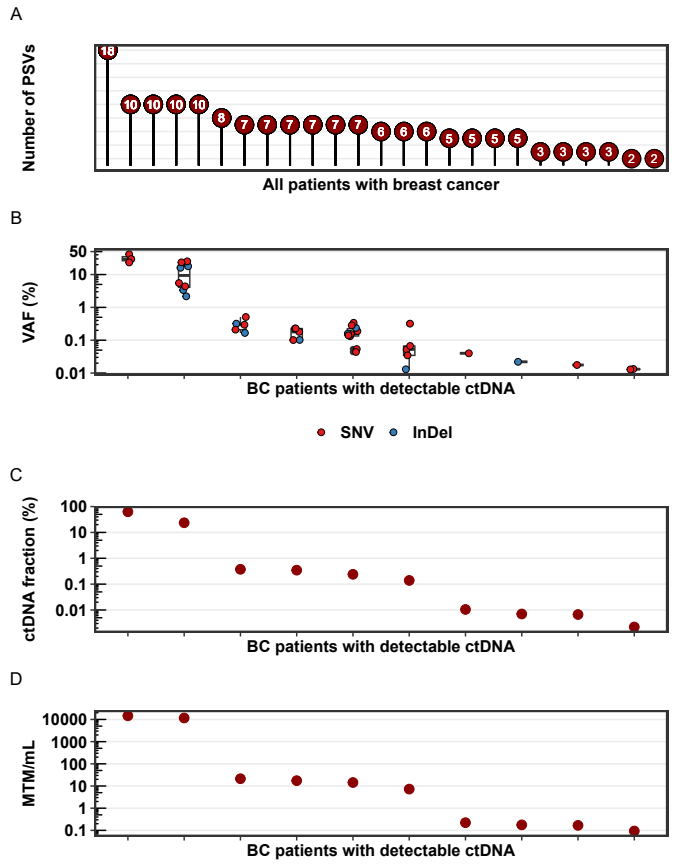


## Fig. S12. Summary of patient-specific variants and tumor burden of breast cancer patients.

(**A**) The number of patient-specific variants incorporated into the personalized panel for each BC patient. (**B**) The ctDNA variant allele frequency (VAF), (**C**) ctDNA fraction and (**D**) mean tumor molecules (MTM/mL) in the first postoperative plasma with detectable ctDNA of BC patients. BC: breast cancer.


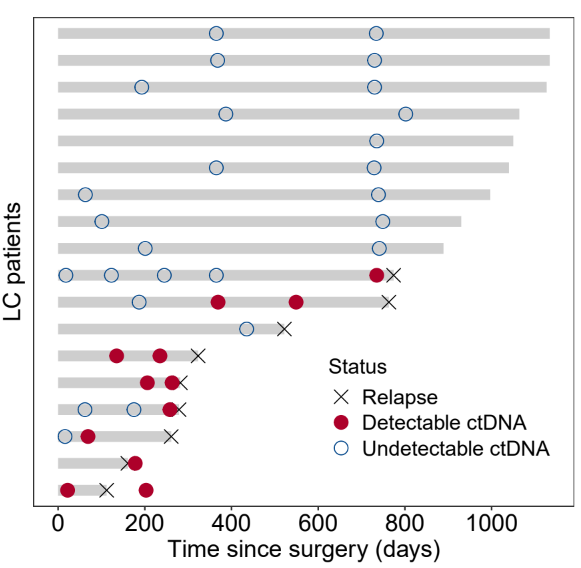


## Fig. S13. Event chart depicting the follow-up history of postoperative MRD testing and disease relapse in lung cancer (LC) patients.


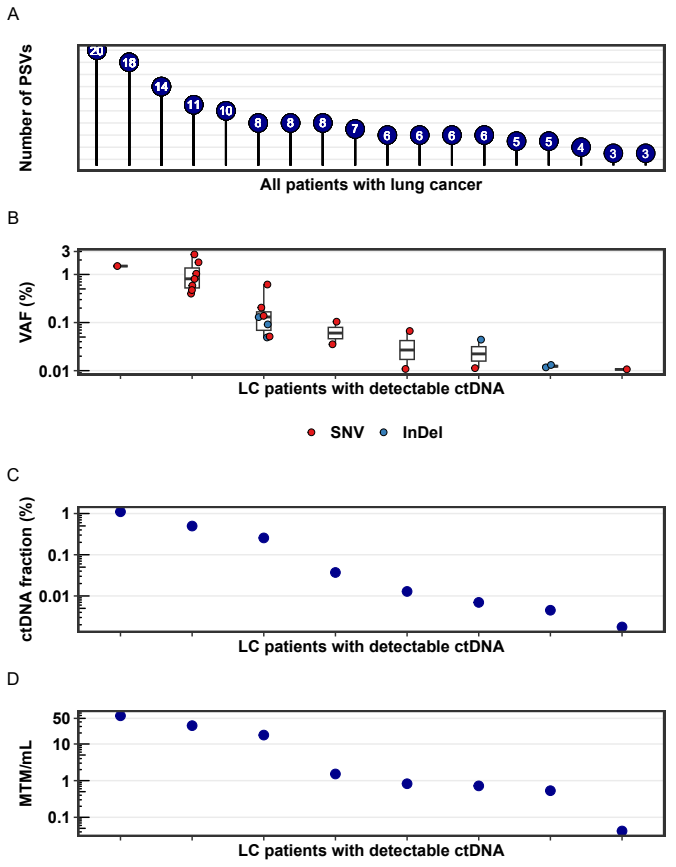


## Fig. S14. Summary of patient-specific variants and tumor burden of lung cancer patients.

(**A**) The number of patient-specific variants incorporated into the personalized panel for each LC patient. (**B**) The ctDNA variant allele frequency (VAF), (**C**) ctDNA fraction and (**D**) mean tumor molecules (MTM/mL) in the first postoperative plasma with detectable ctDNA of LC patients. LC: lung cancer.


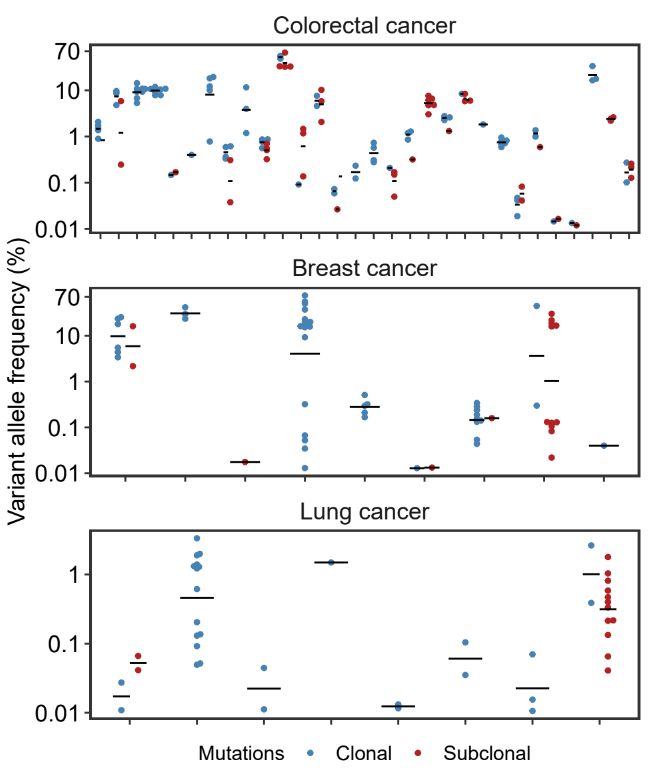


## Fig. S15. The variant allele frequencies of the ctDNA clonal or subclonal mutations in postoperative MRD-positive patients.

Each point represents a detected ctDNA mutation. Crossbars represent the average variant allele frequencies of the ctDNA clonal or subclonal mutations in each patient with detectable minimal residual disease (MRD).

# Supplementary Tables

## Table S1. Mutation list of ctDNA standard sample.

| Gene | Chromosome | cHGVS | pHGVS | Type |
| --- | --- | --- | --- | --- |
| AKT1 | 14 | c.49G>A | p.E17K | SNV |
| ALK | 2 | c.3522C>A | p.F1174L | SNV |
| BRAF | 7 | c.1799T>A | p.V600E | SNV |
| KRAS | 12 | c.38G>A | p.G13D | SNV |
| PDGFRA | 4 | c.2525A>T | p.D842V | SNV |
| KRAS | 12 | c.35G>A | p.G12D | SNV |
| NRAS | 1 | c.35G>C | p.G12A | SNV |
| EGFR | 7 | c.2573T>G | p.L858R | SNV |
| EGFR | 7 | c.2369C>T | p.T790M | SNV |
| EGFR | 7 | c.2582T>A | p.L861Q | SNV |
| KRAS | 12 | c.34G>T | p.G12C | SNV |
| CDK12 | 17 | c.749C>A | p.P250H | SNV |
| IDH1 | 2 | c.394C>G | p.R132G | SNV |
| IDH2 | 15 | c.515G>T | p.R172M | SNV |
| KIT | 4 | c.2447A>T | p.D816V | SNV |
| KRAS | 12 | c.183A>C | p.Q61H | SNV |
| EGFR | 7 | c.2303G>T | p.S768I | SNV |
| NRAS | 1 | c.182A>G | p.Q61R | SNV |
| EGFR | 7 | c.2155G>T | p.G719C | SNV |
| PIK3CA | 3 | c.3140A>G | H1047R | SNV |
| TP53 | 17 | c.818G>A | p.R273H | SNV |
| ERBB2 | 17 | c.2314_2325dup | p.Y772_A775dup | Indel |
| EGFR | 7 | c.2240_2257del | p.L747_P753delinsS | Indel |
| ERBB2 | 17 | c.2313_2324dup | p.Y772_A775dup | Indel |
| EGFR | 7 | c.2310_2311insGGT | p.D770_N771insG | Indel |
| KIT | 4 | c.1504_1509dup | p.Ala502_Tyr503dup | Indel |
| BRAF | 7 | c.1798_1799delinsAA | p.V600K | Indel |
| EGFR | 7 | c.2235_2249del | p.E746_A750del | Indel |
| TPM3:NTRK1 | 1 | N/A | TPM3(7)-NTRK1(10)fusion | SV |
| EML4:ALK | 2 | N/A | EML4(13)-ALK(20)fusion | SV |
| CD74:ROS1 | 5 | N/A | CD74(6)-ROS1(34)fusion | SV |
| CCDC6:RET | 10 | N/A | CCDC6(1)-RET(12)fusion | SV |
| FGFR3:TACC3 | 4 | N/A | FGFR3(17)-TACC3(4)fusion | SV |

## Table S2. Clinical characteristics of 46 patients with colorectal cancer. Related to Figure 5.

| **Characteristics** | **MRD detectable** | **MRD undetectable** | | **P value** |
| --- | --- | --- | --- | --- |
|  | **(N=30)** | **(N=16)** | |  |
| **Age** |  |  |  | |
| Mean (SD) | 56.3 (13.6) | 59.8 (8.90) | 0.311 | |
| Median [Min, Max] | 57.0 [34.0, 84.0] | 57.5 [45.0, 76.0] |  | |
| **Gender** |  |  |  | |
| Female | 14 (46.7%) | 5 (31.3%) | 0.362 | |
| Male | 16 (53.3%) | 11 (68.8%) |  | |
| **Stage** |  |  |  | |
| I | 1 (3.3%) | 5 (31.3%) | 0.0225 | |
| II | 8 (26.7%) | 4 (25.0%) |  | |
| III | 15 (50.0%) | 7 (43.8%) |  | |
| IV | 6 (20.0%) | 0 (0%) |  | |
| **Follow-up time** |  |  |  | |
| Mean (SD) | 530 (380) | 986 (390) | <0.001 | |
| Median [Min, Max] | 462 [65.0, 1650] | 868 [222, 1630] |  | |

Abbreviation: MRD, Molecular Residual Disease; P values of Mann-Whitney test or Fisher's exact test were shown.

## Table S3. Clinical characteristics of 25 patients with triple negative breast cancer. Related to Figure 5.

| **Characteristics** | **MRD detectable** | **MRD undetectable** | **P-value** |
| --- | --- | --- | --- |
|  | **(N=9)** | **(N=16)** |  |
| **Age** |  |  |  |
| Mean (SD) | 44.9 (11.8) | 46.5 (11.4) | 0.744 |
| Median [Min, Max] | 42.0 [32.0, 66.0] | 45.0 [28.0, 73.0] |  |
| **Gender** |  |  |  |
| Female | 9 (100%) | 16 (100%) |  |
| **Stage** |  |  | <0.001 |
| IIA | 1 (11.1%) | 14 (87.5%) |  |
| IIB | 4 (44.4%) | 2 (12.5%) |  |
| IIIA | 2 (22.2%) | 0 (0%) |  |
| IIIC | 2 (22.2%) | 0 (0%) |  |
| **Follow-up time (days)** |  |  |  |
| Mean (SD) | 243 (209) | 727 (199) | <0.001 |
| Median [Min, Max] | 174 [93.0, 742] | 718 [384, 1100] |  |

Abbreviation: MRD, Molecular Residual Disease; P values of Mann-Whitney test or Fisher's exact test were shown.

## Table S4. Clinical characteristics of 18 patients with lung cancer. Related to Figure 5.

| **Characteristics** | **MRD detectable** | **MRD undetectable** | **P-value** |
| --- | --- | --- | --- |
|  | **(N=8)** | **(N=10)** |  |
| **Age** |  |  |  |
| Mean (SD) | 59.3 (11.2) | 61.9 (10.4) | 0.614 |
| Median [Min, Max] | 60.5 [37.0, 70.0] | 59.0 [50.0, 79.0] |  |
| **Gender** |  |  |  |
| Female | 3 (37.5%) | 6 (60.0%) | 0.637 |
| Male | 5 (62.5%) | 4 (40.0%) |  |
| **Stage** |  |  |  |
| I | 3 (37.5%) | 4 (40.0%) | 0.832 |
| II | 2 (25.0%) | 1 (10.0%) |  |
| III | 3 (37.5%) | 5 (50.0%) |  |
| **Follow-up time (days)** | |  |  |
| Mean (SD) | 369 (256) | 989 (184) | <0.001 |
| Median [Min, Max] | 281 [112, 774] | 1050 [522, 1130] |  |

Abbreviation: MRD, Molecular Residual Disease; P values of Mann-Whitney test or Fisher's exact test were shown.
